# Inferring Cognitive Abilities from Response Times to Web-Administered Survey Items in a Population-Representative Sample

**DOI:** 10.3390/jintelligence11010003

**Published:** 2022-12-23

**Authors:** Doerte U. Junghaenel, Stefan Schneider, Bart Orriens, Haomiao Jin, Pey-Jiuan Lee, Arie Kapteyn, Erik Meijer, Elizabeth Zelinski, Raymond Hernandez, Arthur A. Stone

**Affiliations:** 1 Department of Psychology, University of Southern California, Los Angeles, CA 90089, USA; 2 Center for Economic and Social Research, University of Southern California, Los Angeles, CA 90089, USA; 3 Leonard Davis School of Gerontology, University of Southern California, Los Angeles, CA 90089, USA; 4 School of Health Sciences, University of Surrey, Guildford GU2 7YH, UK

**Keywords:** cognitive abilities, response times, survey research, self-report, online surveys, response time variability, population research, inductive reasoning skills, task switching/inhibitory control

## Abstract

Monitoring of cognitive abilities in large-scale survey research is receiving increasing attention. Conventional cognitive testing, however, is often impractical on a population level highlighting the need for alternative means of cognitive assessment. We evaluated whether response times (RTs) to online survey items could be useful to infer cognitive abilities. We analyzed >5 million survey item RTs from >6000 individuals administered over 6.5 years in an internet panel together with cognitive tests (numerical reasoning, verbal reasoning, task switching/inhibitory control). We derived measures of mean RT and intraindividual RT variability from a multilevel location-scale model as well as an expanded version that separated intraindividual RT variability into systematic RT adjustments (variation of RTs with item time intensities) and residual intraindividual RT variability (residual error in RTs). RT measures from the location-scale model showed weak associations with cognitive test scores. However, RT measures from the expanded model explained 22–26% of the variance in cognitive scores and had prospective associations with cognitive assessments over lag-periods of at least 6.5 years (mean RTs), 4.5 years (systematic RT adjustments) and 1 year (residual RT variability). Our findings suggest that RTs in online surveys may be useful for gaining information about cognitive abilities in large-scale survey research.

## 1. Introduction

The monitoring of cognitive abilities in large population surveys is receiving increasing attention in social science, epidemiology, and health policy research. It is widely recognized that cognitive abilities are powerful predictors of important life outcomes, including educational and work performance (Clark et al. 2010; Nye et al. 2022; Robertson et al. 2010; Wai et al. 2018), earnings and financial wellbeing (Furnham and Cheng 2016; Murnane et al. 2000), life satisfaction (Enkvist et al. 2013; St John and Montgomery 2010), health (Luciano et al. 2009; Stilley et al. 2010), successful aging (Castro-Lionard et al. 2011), and mortality (Batty et al. 2007; Duff et al. 2009). Large-scale monitoring of people’s cognitive abilities allows for the investigation of protective and risk factors of cognitive impairment associated with aging (Finkel et al. 2003) and chronic medical conditions (Schagen et al. 2014).

### 1.1. Conventional and Alternative Approaches to Cognitive Testing

Cognitive assessments have traditionally been conducted face-to-face in clinic settings using standardized tests and trained health professionals who oversee their administration (Woodford and George 2007). In this setting, cognitive testing is not routinely and infrequently conducted, which has limited opportunities for tracking intra-individual changes in cognitive abilities (Seelye et al. 2015). For pragmatic reasons, in population survey research cognitive functioning tests have often been conducted over the telephone, especially when in-person assessments were not viable (Lachman et al. 2014; Langa et al. 2009). With the advancement of online data collection, efforts have increasingly been underway to conduct cognitive testing over the internet (Bissig et al. 2020; Feenstra et al. 2018; Tsoy et al. 2021). All of these modes of administration, however, have been shown to be costly, time-intensive, and/or burdensome to respondents.

Recently, alternative approaches to infer individuals’ cognitive abilities from other behaviors have been proposed that do not require the use of cognitive tests and that can overcome some of their practical limitations. The core assumption of these approaches is that unobtrusive monitoring of people’s day-to-day behaviors during routine but cognitively challenging activities can provide pertinent and ecologically valid information about an individual’s cognitive status and change over time. For example, Kaye and colleagues examined the utility of a home-installed activity assessment system consisting of different types of sensors that were installed in older people’s homes and computers that were provided to participants for daily use (Kaye et al. 2011). The activity system was installed for 33 months, on average. The derived metrics included computer usage, time spent outside of the home, walking speed, and overall daily activity. Annual assessments included physical examinations, neuropsychological testing, and questions about health and functioning. Results showed that passive monitoring can give insight into functioning and performance difficulties in near real-time. The study further demonstrated the feasibility of implementing the technology and engaging older adults in its use. Similarly, passively monitored computer mouse movement patterns during routine home computer use have been shown to be sensitive to detecting mild cognitive impairment (MCI) in older adults (Seelye et al. 2015). Older adults who were cognitively intact or had MCI participated in a longitudinal study that examined in-home monitoring technology. Computer mouse movement patterns during a week of routine at-home computer use were derived. Metrics included total mouse moves, movements with greater variability and less efficiency, and movements with longer pauses. All of these metrics were significantly associated with MCI. The results of both of these studies demonstrate the potential of unobtrusive monitoring of routine but cognitively challenging activities.

### 1.2. The Role of Response Times for Measuring Cognitive Abilities

In the present paper, we examine whether participants’ response times (RTs) to questions in online surveys can be used to infer their cognitive abilities. The assessment of RTs has a long history in cognitive testing. For example, RTs are routinely used to measure perceptual speed in standardized cognitive tests, and distributional characteristics of RTs in laboratory-based response latency tasks have been used to measure higher-order cognitive abilities (Kyllonen and Zu 2016). It is important to acknowledge that an individual’s RT does not necessarily have a uniform relationship with cognitive abilities but instead that RTs interact with a respondent’s cognitive skill level and the complexity of the item or task (Goldhammer et al. 2014; Hunt 1980). For tasks that involve more automated, lower-order cognitive processes some research has found a positive relationship between RTs and a respondent’s skill level. In contrast, higher-order tasks that require controlled cognitive processes have shown an inverse relationship between RTs and respondent skill level, suggesting a calibration of response latency with item difficulty (Dodonova and Dodonov 2013; Goldhammer et al. 2014; Naumann and Goldhammer 2017; Naumann 2019). This calibration holds across a range of cognitive tasks and supports a dual process model of cognition (Coomans et al. 2016; Evans and Stanovich 2013). The dual process model suggests a conceptual dichotomy of processes that are either automatic, rapid, and unconscious, or controlled, slow, and conscious. Both automatic and controlled processes are used in a range of tasks including reasoning, judgment, and social decision making (Evans and Stanovich 2013). For example, Dodonova and Dodonov (2013) assessed RTs and response accuracy during a cognitive task that consisted of items with changing difficulty levels. They examined changes in the relationship between respondents’ RT–accuracy, accuracy–ability, and RT–ability as a function of increasing task difficulty. Their results showed that overall respondents with greater cognitive ability had faster RTs and higher accuracy rates compared to respondents with lower cognitive abilities. With increasing item difficulty, the accuracy-ability relationship strengthened, whereas the speed-ability relationship tended to weaken. In another example, Goldhammer et al. (2014) examined whether the “time on task effect” in computer-based reading and problem-solving tasks is moderated by respondent skill level and task difficulty. Results showed that the time on task effect was positive and amplified with greater task difficulty for problem solving tasks. For reading tasks, the opposite results were found. In addition, the positive time on task effect lessened with greater respondent skill level for problem-solving tasks, whereas the negative time on task effect amplified for respondents with greater skill level for reading tasks. In sum, these studies demonstrate that the relationship between RTs and a respondent’s cognitive skills is complex and may differ depending upon the complexity of the task (requiring more controlled versus routine cognitive processing).

To date, very limited research has examined the possibility that RTs captured as a byproduct of online survey responses could be useful to infer people’s cognitive abilities. We set our focus on RTs in online surveys because web-based data collection has become a mainstay of large-scale survey research opening the door to innovative ways to approach the measurement of cognitive abilities apart from standardized cognitive testing. Response latencies to questionnaire items are routinely collected in most online surveys and are already a standard feature of many web-based data collection platforms making them a cost-effective and readily available source of paradata in online studies.

### 1.3. Response Times and Cognitive Abilities in Survey Research

To date, the use of RTs in survey research has focused on evaluating the quality of survey questions (Yan and Tourangeau 2008), identifying which items are effortful and which are not (Lenzner et al. 2010), detecting survey satisficing and participants engaging in careless responding (Meade and Craig 2012; Schneider et al. 2018), and studying survey fatigue during lengthy questionnaire assessments (Galesic and Bosnjak 2009). An important yet understudied topic in survey research is the question of whether completing an online survey may be a particularly good venue for assessing cognitive function. RTs to items in surveys are indeterminate as to whether responses are objectively accurate, unlike those in many cognitive tasks, such as simple or complex RT, memory or executive attention, reading comprehension or information search on the internet. Nevertheless, surveys can be complex and cognitively demanding tasks and there are clear individual differences in response latency to survey items (Park and Schwarz 2012; Tourangeau et al. 2000). Moreover, survey items are often heterogeneous in contents and demands such that respondents need to adjust their attentional focus and adapt their responses as they navigate through different sets of questions. Thus, responding to survey items arguably requires many different cognitive processes, some of which are automatic, but others of which involve higher-level cognitive abilities including attentional control, planning, organization, and mental flexibility. For this reason, we hypothesize that RTs in online surveys might be particularly well suited for inferring respondents’ higher order cognitive abilities.

### 1.4. The Present Study

The goal of the present study was to evaluate whether it is possible to glean information about people’s inductive reasoning skills (quantitative and verbal) and task switching/inhibitory control from their response times to survey questions in a nationally representative online panel study. In order to capture a range of information relevant to higher-order cognitive functioning, we examined multiple variance components inherent in question RTs in the completion of multi-item surveys, including a person’s mean RT and patterns of intraindividual RT variability.

We expected that faster mean RTs for survey items would be associated with greater inductive reasoning skills and task switching/inhibitory control (Schmiedek et al. 2007). Furthermore, we carefully considered the role of intraindividual variability in RTs to survey items. Greater intraindividual RT variability in standardized reaction time tasks has been associated with less efficient neural transmission and lower intelligence (Deary and Caryl 1997; Hanes and Schall 1996; Jensen 1992; Slifkin and Newell 1998). Accordingly, we expected that greater RT variability in survey item responses may also be associated with lower cognitive abilities. However, given the heterogeneous nature of survey items, it is also the case that greater RT variability in online surveys may in part indicate that respondents systematically adjust their RT to the demands of different survey items, which may reflect greater mental flexibility and greater executive functioning (Gehring et al. 1993; Holroyd and Coles 2002). We therefore speculated that two different components of intraindividual RT variability can be distinguished: one reflecting “systematic RT adjustments” (i.e., variability in RTs in response to variation in item demands) and one reflecting “residual RT variability” (i.e., spontaneous RT fluctuations that are not explained by systematic RT adjustments), both of which may be associated with cognitive abilities but in opposite directions.

A second goal was to evaluate prospective associations between RT components in survey item responses and people’s cognitive abilities. Impairments in higher order cognitive functioning are early indicators of neurodegenerative disease and dementia (Gallassi et al. 2002; McKhann et al. 2011). A major advantage of RTs from longitudinal online surveys is that they are available repeatedly over time, potentially facilitating early detection of declines in cognitive abilities. We examined the maximal time lag for which the different RT components in survey item responses would allow for the longitudinal prediction of subsequent scores from standardized cognitive (inductive reasoning skills and task switching/inhibitory control) tests. If it were possible to gain information about a respondent’s cognitive abilities from their RTs in web-based surveys, this would set the stage for larger scale monitoring of cognitive abilities in the general population in addition to standard cognitive testing.

## 2. Materials and Methods

### 2.1. Participants

The data analyzed were drawn from the Understanding America Study (UAS), a probability-based internet panel initiated in 2014 (Alattar et al. 2018). The panel is housed at the University of Southern California and currently has ~10,000 adult panel members. In contrast to convenience (opt-in) panels, UAS panel members are recruited through nation-wide address-based sampling, which tends to reduce many biases in population parameters estimated from convenience panels where members self-select to participate (Yeager et al. 2011). UAS panelists without internet access are equipped with a tablet and broadband internet to achieve representativeness, given that internet access tends to be lower among older and less educated Americans (Couper et al. 2007). As is typical for large-scale internet panels, UAS respondents complete about 1–2 web-based surveys per month. Response rates are routinely high (75–95%), and attrition rates are modest (7–8% per year).

### 2.2. Survey and Item Selection

Survey items were drawn from 42 UAS surveys administered between 2014 and 2021 (administered on average in about 2-month intervals) on a wide variety of topics, including perceived wellbeing, retirement planning, financial decision making, personality, and health behaviors (for an overview of survey contents see https://uasdata.usc.edu/, accessed on 12 December 2022). Most surveys cover more than one topic. Since respondents entered the UAS at different times (the panel is still growing), the number of surveys for which RTs were available as paradata differed across respondents. For each respondent, only surveys administered before each of the formal cognitive tests (see below) were included, and the analysis included only UAS respondents who had at least 5 of the 42 surveys completed at the time of analysis.

Within each of the UAS surveys, items were eligible for the analysis regardless of their content, however, we specified several criteria for item inclusion: (1) items needed to be shown individually on a page because the RT timestamps recorded as paradata in the UAS were recorded per page; this excluded survey items presented together on the same screen in grid or matrix format; (2) open-ended questions were excluded; and (3) 75% of respondents or more needed to have completed an item to reduce potential selection biases for items involving skip patterns (item nonresponse tends to be low in the UAS, but skip patterns are relatively common). The mean number of items analyzed per survey was 28.34 (median = 25 items, SD = 14.92, range = 10 to 65 items); a total of 1173 survey items were included (for a sample of 50 survey items illustrating the heterogeneity of item contents, see Table S1 in the Supplementary).

### 2.3. Recording of Response Times

The UAS administers surveys using the NubiS data collection tool. NubiS creates HTML based question screens and sends these to the browser for display. Respondents provide their answers on their computers, tablets, or smartphones using navigational buttons to move through the survey. The NubiS tool uses Hypertext Preprocessor code to record RTs as the number of seconds spent on each question screen, defined as the moment from which NubiS sends a question screen to the browser to the moment it receives a signal that the respondent has exited the screen. RTs encompass respondents’ reading and answering time and excludes any time on the server for processing answers and creating question screens. For each survey item, RTs were trimmed at the 99th percentile of respondents to eliminate extreme outliers (e.g., respondents stepping away from their computer) (Ratcliff 1993). RTs were log transformed as is customary to normalize the distribution of RT data (Thissen 1983). Henceforth, we refer to the log-transformed RTs as RTs for simplicity.

### 2.4. Online Cognitive Tests

#### 2.4.1. Quantitative Reasoning

The Number Series task was used to measure quantitative reasoning, a type of inductive reasoning skill that involves the ability to solve problems that depend upon mathematical relationships (Mather and Jaffe 2016). Respondents are presented a series of numbers with one number missing from the series (e.g., 4, 7, 10, ?). The task is to determine the numerical pattern in the series and to provide the missing number. The UAS administers Number Series items that had previously been implemented as self-administered online tests in the Cognition and Aging in the USA (CogUSA) study (McArdle et al. 2015), in 2-year intervals. Two parallel forms with 15 items each are rotated across biennial assessments to reduce practice effects. For participants who had completed the online task more than once, the last assessment completed was used in the present analyses. Items are scored using Item Response Theory (IRT) using Samejima’s Graded Response Model (Samejima 1969), and test scores are scaled in T-scores, where 50 is the mean and 10 is the SD of a census-weighted sample of the general adult US population. Higher scores indicate better quantitative reasoning.

#### 2.4.2. Verbal Reasoning

The Verbal Analogies task was used to measure verbal reasoning, an inductive reasoning skill involving the comprehension of concepts expressed through language (Mather and Jaffe 2016). In this task, respondents need to recognize a relationship between two words and successfully apply it to two other words (e.g., “Night” is to “Dark” as “Day” is to ?). The UAS also administers Verbal Analogies from the CogUSA (McArdle et al. 2015) study as online self-administered test, where two 15-item parallel forms are counterbalanced across occasions. For each participant, the last assessment was analyzed in the present study. The test is scored using IRT, with scores normed on a T-score metric (mean = 50 and SD = 10 in the general adult US population); higher scores indicate better verbal reasoning skills.

#### 2.4.3. Task Switching/Inhibitory Control

We used the Stop-and-Go Switch task as a measure of task switching and inhibitory control (Lachman et al. 2014). Participants are presented with the word red or green and are asked to respond with either stop or go (i.e., for the color red respond with stop; for the color green respond with go). The test includes three conditions (baseline, reverse baseline, mixed conditions) that are administered sequentially and that include reversals of the instructions (i.e., stop for green; go for red). A switch trial is defined as the first response after a participant is asked to change from one instruction to another. A nonswitch trial does not involve a change in instructions. The Stop-and-Go Switch task was originally developed for telephone administration, implemented in the Midlife in the United States National Longitudinal Study (MIDUS) (Lachman et al. 2014; Tun and Lachman 2008). The UAS developed an adapted version for self-administered web administration that has been validated in prior research (Liu et al. 2022). Latencies were measured, in milliseconds lapsed, between the presentation of the cue and the correct response. Participants needed to have at least 70% correct trials to be scored, which was deemed an acceptable threshold to exclude respondents with invalid or careless answer behavior (Liu et al. 2022). The baseline conditions are administered to measure choice reaction time and were not examined here. Latencies in the mixed condition are considered an assessment of task switching and inhibitory control (Lachman et al. 2014). We followed the scoring procedures used in MIDUS: median latencies were first calculated for the switch and nonswitch trials of the mixed condition in order to eliminate the effects of outliers, and the average of the median latencies for switch and nonswitch trials was used as a measure of task switching/inhibitory control (Hughes et al. 2018; Lachman et al. 2014). For the present analyses, the median latencies were reverse scored such that higher scores on the variable indicate better functioning

### 2.5. Data Analysis

The analyses were conducted in multiple steps. First, we derived measures of RT components (mean and intraindividual variability in each person’s RTs) from the log-transformed survey item RT data. Second, we examined associations between the derived RT measures and participants’ cognitive abilities. The third step examined the temporal stability of their relationships between the RT measures and subsequent tests of cognitive abilities.

#### 2.5.1. Step 1: Deriving Survey Item RT Component Measures

We used a multilevel structural equation modeling (MSEM) approach to calculate the RT component measures. MSEM accommodates the nested structure of the RT data, with RTs for multiple survey items nested within respondents, accounts for measurement error in observed RTs, and has proven useful for capturing intraindividual RT dynamics and quantitative differences in these dynamics between individuals (Hamaker and Wichers 2017; McNeish and Hamaker 2020). Two different models were estimated in an attempt to isolate relevant RT component measures from respondents’ RT patterns.

Our first model was a so-called “location-scale” multilevel model, an extension of the traditional multilevel model that allows for random effects (i.e., individual differences) in means or intercepts (referred to as “location” in statistical terms) and in intraindividual variability (referred to as “scale”) in the same model (Hedeker et al. 2012; McNeish and Hamaker 2020). The model captures respondents’ average RT and amount of intraindividual variability in RTs as two latent variables. Based on prior research on RT variability in standardized (laboratory-based) reaction time tests, we hypothesized that slower average RTs and greater intraindividual RT variability would be associated with worse cognitive functioning abilities (Haynes et al. 2017; Rutter et al. 2020; Tam et al. 2015). The location-scale model can be described with the following multilevel equations:

Level 1: *RT_ij_* = *α_j_* + *r_ij_*,

where *r_ij_* ~ *N*(0,*σ_j_*^2^)

Level 2: *α_j_* = *γ*_00_ + *u*_0*j*_ (1)

       log(*σ_j_*^2^) = ω + *u_1j_*,

where $\left(\begin{array}{c} u_{0j}\\ u_{1j} \end{array}\right)\sim MVN\left(\left(\begin{array}{c} 0\\ 0 \end{array}\right),\left(\begin{array}{cc} \tau_{00} & \;\\ \tau_{10} & \tau_{11} \end{array}\right)\right)$.

At Level 1 (the within-person level), the observed (log-transformed) response time RT for item *i* and participant *j* equals the sum of a person-specific mean RT *α_j_* and a residual deviation from that mean RT, *r_ij_*. The residual deviations are assumed to follow a normal distribution with mean 0 and variance *σ_j_*^2^. In contrast to traditional multilevel models, where the variance of these residuals is assumed to be the same for all individuals, the location-scale multilevel model allows this variance to differ between individuals (as indicated by the individual-specific subscript *j*). Level 2 (the between-person level) captures these individual differences as random effects (i.e., latent variables). The random effects in person-specific RT means *u*_0*j*_ and in the log of each person’s intraindividual RT variance *u*_1*j*_ are assumed to follow a multivariate normal distribution with mean vector 0 and covariance matrix *τ* (McNeish and Hamaker 2020).

Our second model was an expanded version of the location-scale model, based on the assumption that different components of intraindividual RT variability can be distinguished from each other that may relate to cognitive abilities in opposite ways. Specifically, survey items vary considerably in difficulty and the cognitive demands associated with them (Schneider et al. forthcoming). In RT modeling, a concept analogous to item difficulty is the “time intensity” of an item, defined as “the amount of time an item tends to require” (Kyllonen and Zu 2016, p. 14). We speculated that one component of intraindividual RT variability consists of systematic adjustments whereby a person adjusts their RTs to the time intensity levels of the items. Such RT adjustments may reflect greater responsiveness to changing stimuli and greater mental flexibility, which are important aspects of fluid intelligence and executive functioning (Gehring et al. 1993; Holroyd and Coles 2002). This component may be distinguished from residual intraindividual RT variability that is unrelated to the time intensity of the items and might reflect attentional lapses and neural noise (Deary and Caryl 1997). Accordingly, the second model expanded the location-scale model by distinguishing systematic RT adjustments and residual RT variability as two between-person latent variables:

Level 1: *RT_ij_* = *α_j_* + *β_j_*
*TI_i_* + *r_ij_*,

where *r_ij_* ~ *N*(0,*σ_j_*^2^)

Level 2: *α_j_* = *γ*_00_ + *u*_0*j*_ (2)

     *β_j_* = *γ*_10_ + *u*_1*j*_

       log(*σ_j_*^2^) = ω + *u*_2*j*_,

where $\left(\begin{array}{c} u_{0j}\\ u_{1j}\\ u_{2j} \end{array}\right)\sim MVN\left(\left(\begin{array}{c} 0\\ 0\\ 0 \end{array}\right),\left(\begin{array}{ccc} \tau_{00} & \; & \;\\ \tau_{10} & \tau_{11} & \;\\ \tau_{20} & \tau_{21} & \tau_{22} \end{array}\right)\right)$.

At Level 1 of the expanded model, the observed (log-transformed) response time for item *i* and participant *j* is regressed on the time intensity (TI) for item *i*, such that *RT_ij_* equals the sum of a person-specific intercept *α_j_*, the item’s time intensity multiplied by a person-specific slope parameter *β_j_*, and a residual *r_ij_*. Consistent with prior RT models (Kyllonen and Zu 2016; van der Linden 2006), we obtained the TIs of the survey items from a cross-classified multilevel model of the log RTs with crossed random effects on subject- and item-levels, where the item-level random effect indicates latent differences in TIs between items. Estimated TIs were saved and centered at 10 s (approximately the average TI across all items, see Section 3) when entered in the expanded location-scale model. This means that the intercept of the expanded model captures the person’s predicted RT for an item with a TI of 10 s (which we refer to as a person’s mean RT thereafter), the slope captures the predicted increase and decrease in the person’s RT for items with higher and lower TIs (which we refer to as “systematic RT adjustments”), and the residual captures the deviations of the observed RT from the predicted RT for each person and item (referred to as “residual RT variability”). At Level 2, random effects represent latent individual differences in mean RTs *u*_0*j*_, in systematic RT adjustments *u*_1*j*_, and in residual RT variability *u*_2*j*_. All multilevel models were estimated in M*plus* version 8.8 (Muthén and Muthén 2017) using Bayesian parameter estimation with software default diffuse priors. A graphical representation of the MSEM approach to estimating the latent variables involved in the expanded version of the location-scale model is shown in Figure S1 (for M*plus* code, see Figure S2 in the Supplementary).

#### 2.5.2. Step 2: Associations between RT Component Measures and Cognitive Abilities

The RT component measures described above were estimated based on all survey response times from all included UAS surveys available for each person prior to each of the cognitive assessments. To examine the relationships between the RT component measures and respondents’ cognitive abilities, we examined bivariate correlations and performed multiple regressions in which the RT component measures served as multiple predictor variables and a cognitive functioning variable (quantitative reasoning, verbal reasoning, and task switching/inhibitory control, in separate models) served as dependent variable. Additional multiple regressions controlled for demographics of age, gender, race, ethnicity, education, and income, entered as covariates. We also explored whether the relationships between the RT component measures and cognitive abilities differed between younger (less than 40 years of age) and relatively older (40 years or older) participants using moderated regression models with age as a moderator. Separate regression models were estimated for the RT measures derived from the location-scale model and from the expanded location-scale model.

#### 2.5.3. Step 3: Stability of Lagged Relationships with Cognitive Abilities

To examine the temporal stability of prospective (i.e., lagged) relationships between the RT component measures and cognitive functioning outcomes, we first determined the time intervals between the administration of each of the 42 surveys and the time point of cognitive testing (i.e., lag times) for each participant. Next, we estimated the (expanded) multilevel location-scale model separately for each of the 42 surveys and created an average score of each RT component measure for every half-year interval before the cognitive test (i.e., 0–0.5 years, >0.5–1 years, and so on, up to >6–6.5 years before the test). We then estimated the lagged associations between the RT component measures and each specific cognitive test for increasingly longer lag times, using bivariate correlations and multiple regression models (entering the RT components in combination as predictors of each cognitive test). To compare the regression coefficients of the RT components across the different lag times, we used a “lag as moderator” approach (Selig et al. 2012) whereby the RT components for all lag time periods were used as time-varying predictors of the cognitive scores and the RT component by lag time (used as categorical variable) interactions were tested. Regression analyses were conducted using the SURVEYREG procedure in SAS 9.4 (Cary, NC, USA) with cluster-robust standard errors.

The maximum time lag for which the RT components were significantly associated with cognitive test scores was determined by inspecting the longest consecutive lag for which the 95% confidence interval of the regression coefficient did not include 0. To quantify the magnitude of relationships between the RT component measures and cognitive test scores, we considered correlations and standardized regression coefficients of .10, .30, and .50 as small, medium, and large effects, respectively (Cohen 1988).

## 3. Results

### 3.1. Descriptive Characteristics

The demographic characteristics of the participant sample are presented in Table 1. The sample composition differed somewhat across analyses for the three cognitive tests because UAS respondents joined the panel and completed the cognitive assessments at various time points. Across all three samples, the majority of respondents were between the ages 18 and 54 years, and about one third were between the ages of 55 and 74 years. The racial and ethnic composition of respondents was largely representative of the general US population. Slightly more than half of the respondents were female. With regard to educational attainment, respondents with high school graduation or less comprised about a quarter of the sample, respondents with some college education comprised about one third of the sample, and respondents with a college degree comprised almost half of the sample. For annual household income, slightly less than half of the respondents had incomes up to $49,999, about one quarter had incomes up to $99,999, and about a quarter had incomes of $100,000 or more.

The number of UAS surveys and items included in the analyses also differed by cognitive test. For number series, the average number of surveys per respondent was 19.76 (SD = 7.48, range = 5 to 36 surveys) and the average number of items per respondent was 548.10 (SD = 194.25, range = 84 to 1109 items). For verbal analogies, the average number of surveys was 19.57 (SD = 8.09, range = 5 to 37) and the average number of items was 549.26 (SD = 204.96, range = 85 to 1109 items). For the stop-and-go switch task, the average number of surveys was 21.51 (SD = 9.25, range = 5 to 38) and the average number of items was 572.10 (SD = 247.96, range = 85 to 1064 items). In total, 5,004,187 survey item RTs were analyzed for number series, 5,052,106 item RTs for verbal analogies, and 4,362,812 item RTs for the stop-and-go switch task.

**Figure 1:**
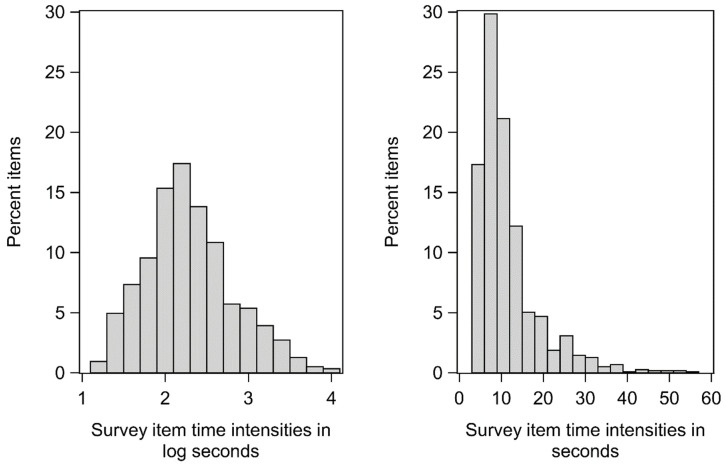
Distribution of time intensities of the survey items (n = 1173) in log seconds (**left**) and in seconds (**right**). Time intensities in log seconds were used in the analyses.

The mean log RT across all participants and survey items was 2.33 log seconds (SD = 0.79, range = .00 to 6.38 log seconds) for analyses involving the number series test, 2.33 log seconds (SD = 0.79, range = .00–6.38 log seconds) for verbal analogies, and 2.32 log seconds (SD = 0.78, range = .00–6.38 log seconds) for the stop-and-go switch task. Item TIs (i.e., the expected item-level RTs) were calculated from all available RTs and are therefore not specific to each cognitive test. The distribution of the item TIs is shown in Figure 1 (in log seconds and back-transformed median seconds per item). The mean of the items’ TIs was 2.29 log seconds (11.53 s when back-transformed), with a range of 1.14 to 4.04 log seconds (3.14 to 57.00 s when back-transformed).

Scores from the three cognitive tests showed moderate to large positive intercorrelations. Number Series and Verbal Analogies tests correlated at *r* = .64 (*p* < .001). Stop-and-Go task scores correlated *r* = .22 (*p* < .001) with Number Series and *r* = .23 (*p* < .001) with Verbal Analogies scores.

### 3.2. Prediction of Cognition from RT Components Derived from the Location-Scale Model

The two RT components derived from the location-scale model were each participant’s mean RT and each participant’s RT variability (whereby intraindividual RT variability was not decomposed into subcomponents) across survey items. The two RT components were weakly correlated with each other (*r* = −.03, *p* = .01, for the Number Series sample, *r* = −.03, *p* = .004, for the Verbal Analogies sample, *r* = −.04, *p* = .002, for the Stop-and-Go Switch task sample).

Table 2 shows the correlations between the RT components and cognitive tests, and results from multiple regressions in which the RT components served as multiple predictors of each cognitive test. As expected, higher mean RTs were significantly negatively associated with each of the cognitive tests, indicating that people with slower mean RTs had lower cognitive scores. Standardized regression coefficients were small in magnitude for Number Series (β = −.06) and Verbal Analogies (β = −.16), and medium to large for the Stop-and-Go Switch task (β = −.41). Contrary to our expectation, greater intraindividual variability in RTs was significantly positively associated with each of the cognitive tests with small effect sizes (regression coefficients ranging from β = .14 for Verbal Analogies and the Stop-and-Go Switch task to β = .19 for Number Series, *p*s < .001), indicating that more variable RTs were predictive of higher cognitive scores. The two RT components in combination explained 4% (Number Series), 5% (Verbal Analogies) and 19% (Stop-and-Go Switch task) of the variance in the cognitive test scores. The pattern of results remained similar after demographic covariates were controlled; however, the relationship between mean RTs and Number Series scores became nonsignificant (see Table 2).

As shown in Table 3, the relationships between a person’s mean RTs and the cognitive test scores were significantly stronger (i.e., more negative) for older (40+ years of age) compared to younger (less than 40 years of age) participants. Specifically, for Number Series and Verbal Analogies, the negative relationships between mean RTs and cognitive test scores were only evident among older participants (β = −.17 and −.32, respectively) but were nonsignificant among younger participants (β = .03 and .01, respectively). For the Stop-and-Go Switch task, even though mean RTs were significantly negatively associated with Stop-and-Go Switch task scores in both age groups, the association was significantly more pronounced at older ages (β = −.48) compared to younger ages (β = −.25). Relationships between RT variability and cognitive test scores did not show pronounced age differences (for Number Series, the association was significantly more pronounced among older compared to younger participants, *p* = .01, but the difference in standardized regression coefficients was small; Table 3).

### 3.3. Prediction of Cognition from RT Components Derived from the Expanded Location-Scale Model

In the expanded location scale model, in addition to deriving an estimate of respondents’ mean RT, intraindividual RT variability was decomposed into subcomponents where one component represented the variation in RT with variation in item TIs (“systematic RT adjustments”) and the second component reflected variation in RTs that were unrelated to variation in the TIs of the items (“residual RT variability”). The three RT components were moderately intercorrelated: mean RTs and systematic RT adjustments were positively correlated at *r* = .29 (for the Verbal Analogies sample) to *r* = .38 (for the Stop-and-Go Switch task sample); mean RTs and residual RT variability were negatively correlated at *r* = −.27 (for the Number Series sample) to *r* = −.31 (for the Stop-and-Go Switch task sample); systematic RT adjustments and residual RT variability were negatively intercorrelated at *r* = −.09 (for the Verbal Analogies sample) to *r* = −.11 (for the Stop-and-Go Switch task sample), all *p*s < .001. The modest size of these correlations among the RT components indicated no multicollinearity problems when entering them simultaneously as predictor variables in regression models.

Table 4 shows the correlations between these RT components and the cognitive tests, as well as results from multiple regressions predicting each cognitive test from these RT components in combination. Slower mean RTs were significantly negatively associated with each of the cognitive tests with medium to large effect sizes (standardized regression coefficients ranging from β = −.28 for Number Series to β = −.48 for the Stop-and-Go Switch task, *p*s < .001). As hypothesized, distinguishing between systematic RT adjustments and residual RT variability yielded effects of intraindividual variability in opposite directions. More pronounced RT adjustments were significantly positively associated with each of the cognitive tests, with large effects for Number Series (β = .50, *p* < .001) and Verbal Analogies (β = .42, *p* < .001) and a small to medium effect for the Stop-and-Go Switch task (β = .22, *p* < .001). Greater residual RT variability was negatively associated with Number Series (β = −.15, *p* < .001) and Verbal Analogies (β = −.16, *p* < .001) scores, with small effects in the expected direction; unexpectedly, greater residual RT variability was very weakly positively associated with performance on the Stop-and-Go Switch task (β = .04, *p* < .001). The three RT components in combination explained 26% (Number Series), 22% (Verbal Analogies) and 22% (Stop-and-Go Switch task) of the variance in the cognitive test scores.

The pattern of results remained similar after demographic covariates were controlled (see Table 4). However, the effect of the residual RT variability component on the Stop-and-Go Switch task became very weakly negative (β = −.04, *p* < .001), consistent with the originally predicted direction of the effect.

Moderator analyses by age showed that the relationships between mean RTs and the cognitive test scores were significantly more negative for older compared to younger participants. As shown in Table 5, the effects of mean RTs were between 1.5 and 2 times larger among older (βs ranging between −.30 and −.53) compared to younger (βs ranging between −.19 and −.25) participants. No significant age differences were evident for the relationships of the cognitive tests with people’s systematic RT adjustments or with residual RT variability, respectively.

### 3.4. Stability of Lagged Relationships with Cognitive Abilities

We next present results for the lagged associations between RT component measures and the cognitive tests for increasingly longer lag times. Given that the RT components from the location-scale model produced only weak effects as shown above, we limit the presentation to results involving RT components from the expanded location-scale model (for results from the location-scale model, see Figures S3 and S4 in the Supplementary).

**Figure 2:**
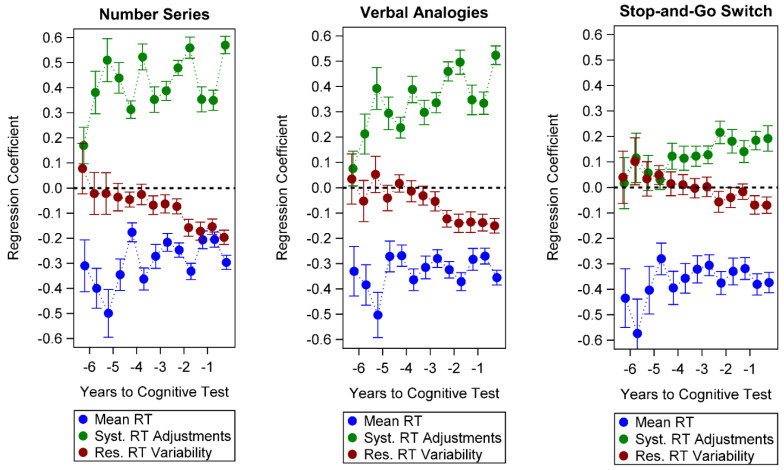
Standardized regression coefficients for the prediction of cognitive test scores from time-lagged survey item response time (RT) components derived from the expanded location-scale model. Error bars represent 95% confidence intervals. Syst. = systematic; Res. = residual.

Lagged effects in half-year intervals before each cognitive test are shown in Table 6, Table 7 and Table 8 (with tests of overall model fit, main effects of RT components, and interactions by lag period) and graphically illustrated in Figure 2. Slower mean RTs prospectively predicted lower scores on each of the three cognitive tests over the full lag period of 6.5 years with small to medium effects; the magnitude of the associations significantly differed across lag periods (*p* < .001 for all mean RT by time-period interactions) but did not show clear monotonic trends for increasingly longer time lags for any cognitive test.

The effects of systematic RT adjustments significantly varied across time lags (*p*s < .001) with decreasing trends in the magnitude of associations with the cognitive tests. More pronounced RT adjustments predicted significantly better Number Series scores for a lag period of up to 6 years with consistently medium effects; a small statistically significant effect was evident for a lag period of 6.5 years. The effect of systematic RT adjustments in predicting Verbal Analogies steadily decreased in magnitude from medium/large to small effects over the years, but remained significant for up to 6 years. Finally, systematic RT adjustments significantly predicted better scores on the Stop-and-Go Switch task, with generally small effects, for a lag period of up to 4.5 years.

Finally, the effects of residual RT variability were weak in magnitude and less temporally stable (*p*s < .001 for residual RT variability by time period interactions); greater residual RT variability predicted significantly worse scores on Number Series over a period of up to 3.5 years, on Verbal Analogies over 3 years, and on the Stop-and-Go Switch task over a period of 1 year, with small to very small effect sizes. The pattern of results remained similar with overall weaker effects of the mean RT component after demographic covariates were controlled (see Figure S5 in the Supplementary).

### 3.5. Summary of the Results

In sum, our results showed that the RT components derived from the initial location-scale model explained only very little proportions of the variance (between 4 and 5%), whereas the RT components derived from the expanded location-scale model explained between 22 and 26% of the variance in each of the three cognitive tests. Out of the three RT components considered in the expanded model, the strongest relationships with cognitive test scores were evident for respondents’ mean RTs (especially among older respondents and for task switching/inhibitory control) and systematic RT adjustments (especially for inductive reasoning tests), whereas the residual RT variability showed weak relationships with cognitive tests. The RT components further demonstrated moderate stability in prospective associations with cognitive assessments.

## 4. Discussion

Surveys are ubiquitous in research and clinical practice. The usefulness of paradata from online surveys for inferring respondents’ cognitive abilities has received little scientific attention. We examined whether response times (RTs) to survey items could be used to infer inductive reasoning skills and task switching/inhibitory control in a large probability-based longitudinal internet panel. Because little is known to date about which specific RT components in survey responses may be most relevant to cognitive functioning, we explored the utility of two different models to derive information about a person’s mean RT and patterns of intraindividual RT variability.

### 4.1. Success of the Location-Scale Model RT Components

The RT components derived from the first model, a multilevel location-scale model that captures individual differences in mean RTs and in intraindividual RT variability, explained only little (4% to 5%) variance with small effect sizes in the inductive reasoning measures (numerical and verbal reasoning scores) and 19% of the variance (a medium to large combined effect) in task switching/inhibitory control scores. Contrary to our hypothesis, a greater amount of intraindividual variability in RTs across survey items showed small positive associations with higher cognitive abilities. This result may seem counterintuitive and surprising in light of a substantial body of research that has viewed greater intraindividual RT variability in elementary speed tasks as detrimental to the successful solving of complex intelligence test items (Jensen 1992; Joly-Burra et al. 2018; Rammsayer and Troche 2010; Schmiedek et al. 2007; Schulz-Zhecheva et al. 2016) and as associated with developmental cognitive decline (Haynes et al. 2017). However, it is also the case that, contrary to the expanded model discussed below, the “naïve” location-scale model did not distinguish between components of intraindividual variability that could either be attributable to inconsistencies in response speed (e.g., neural “noise”, which has often been found to be related to lower cognitive abilities) (Haynes et al. 2017) and systematic adjustments of the speed of responding to variations in the task demands (e.g., switching strategies in accordance with the demands of different survey items, which is related to better cognitive abilities) (Hunt 1980).

In view of the small positive correlations between intraindividual RT variability and cognitive test scores, it is possible that the variability measure predominantly captured people’s ability to adapt their response speed to differing task demands associated with the survey items. Arguably, however, conflating these two aspects of intraindividual RT variability in responses to survey items yields an ambiguous blend of RT components that relate to cognitive functioning in opposite directions. This highlights that results from experimental RT research based on simple choice reaction time tasks and other elementary speed tasks may not directly translate to RTs found in survey settings and that ignoring the differences between the tasks yields largely uninterpretable results. This conclusion further aligns with prior research that has shown that the relationship between RTs and a respondent’s cognitive skills is not necessarily uniform but is instead a function of whether a task requires more controlled, higher-order cognitive processes (e.g., problem-solving) or more routine automated cognitive processes (e.g., reading) (Dodonova and Dodonov 2013; Goldhammer et al. 2014; Naumann and Goldhammer 2017; Naumann 2019).

### 4.2. Success of the Expanded Location-Scale Model RT Components

Our second model used an expanded version of the multilevel location-scale model in an attempt to explicitly acknowledge that survey items vary considerably in the cognitive demands—operationalized as time intensity (TI) differences—associated with them. We argued that an individual’s item-to-item RT variability can be separated into two distinct components: (a) systematic RT adjustments whereby a person adjusts their response speed to the TI levels of the items, and (b) residual intraindividual RT variability that might be attributed to random noise in responding. This distinction is in line with Fiske and Rice’s (1955) seminal early work that stressed the importance of considering different types of short-term fluctuations including Type III variability, which they defined as “variability in response with variation in the stimulus or in the situation” (p. 236) and Type I variability, defined as spontaneous variability that is not a response to stimulus variation.

As expected, the TIs of the items in the UAS surveys varied widely, from 3 s to about 60 s across items. When the item TIs were incorporated as predictors in the expanded multilevel location-scale model, the resulting RT component variances together explained between 24% and 26% variance in the inductive reasoning measures and 22% of the variance in task switching/inhibitory control, a considerable improvement over the RT components derived from the original location-scale model. While these represent large effect sizes by common conventions (Cohen 1988), the proportions of variances explained are of course not near values that would suggest that the RT components can be viewed as interchangeable with any of the three cognitive tests. As discussed next, however, all 3 RT components from the expanded location-scale model showed unique associations with the cognitive test scores in theoretically expected directions.

We found that slower mean RTs derived from the expanded model showed moderate negative associations with cognitive functioning that consistently exceeded those from the original location-scale model in magnitude. A likely reason for this is that the expanded model controlled for differences in TIs across surveys, that is, the mean RTs represented the person’s RT for an item with a TI of 10 s. As such, the expanded model may have been better able to capture individual differences in average response speed under conditions that would have been expected if the survey items were homogeneous or interchangeable in time intensity (and perhaps in underlying cognitive demands). In prospective analyses, mean RTs derived from the expanded location-scale model remained robust over a time period of more than 6 years, suggesting that the mean RTs captured relatively stable, “trait-like” individual differences relating to higher-order cognitive abilities.

Although mean RTs showed expected relationships with all three cognitive tests, they showed the strongest relationship with the Stop-and-Go Switch task, both in the original as well as the expanded location scale model. This finding is noteworthy as it points to the differences in cognitive functions that were assessed with the three cognitive tasks. The Stop-and-Go Switch task, as a measure of task switching/inhibitory control, appears to capture somewhat lower-order and more automated cognitive functions compared to the quantitative and verbal analogies tests that assess inductive reasoning, a more complex task that requires more controlled higher-order cognitive functions. Prior research has shown a positive speed-accuracy relationship for lower-order abilities, such as reading speed and attention (Goldhammer et al. 2014; Naumann and Goldhammer 2017). This suggests that mean RTs might more strongly relate to more automated and routine cognitive functions and tasks. It should also be noted that out of the three cognitive tests, the Stop-and-Go Switch task was the only one that was itself based on RTs, whereas the Number Series and Verbal Analogies tests are based on the accuracy of responses.

The second RT component of the expanded location-scale model, labeled systematic RT adjustments, explicitly considered the extent to which individuals’ RTs varied with the TI levels of the items. Greater systematic RT adjustments in response to changing TI levels were positively associated with greater cognitive abilities for all three tests. Among the different RT components, this component had the strongest associations with the inductive reasoning scores, with large effect sizes. These results are in line with prior research showing that the time spent on a given task might be moderated by the difficulty of the task and a respondent’s skill level (Dodonova and Dodonov 2013; Goldhammer et al. 2014). Specifically, studies have shown a positive correlation between a respondent’s skill level (determined through success on a task) and RTs for more complex tasks, such as reasoning (Goldhammer and Entink 2011; Klein Entink et al. 2009). In contrast, for tasks involving basic skills, such as reading, a negative correlation was observed (Richter et al. 2012). Our results of stronger associations with tests involving inductive reasoning compared to task switching/inhibitory control further corroborate these prior studies and suggest that systematic RT adjustments might be uniquely suited for better understanding higher-order, controlled cognitive processes where taking more time on a task or item is not only expected but might yield better (more accurate) results.

Moreover, we found that respondents’ RT adjustment scores showed stable prospective relationships with subsequent cognitive functioning scores over 4 or more years, in line with prior literature suggesting that strategic adjustments in response caution are reliable person-characteristics that are replicable across time and tasks (Hedge et al. 2019).

The third RT component of the expanded location-scale model reflected a respondent’s residual intraindividual RT variability not accounted for by systematic RT adjustments. In contrast to the original location-scale model, where greater intraindividual RT variability related to higher cognitive scores, higher levels of residual RT variability were associated with lower scores on the inductive reasoning tests (and with task switching/inhibitory control after controlling for demographic covariates), consistent with the idea that more random variation in thinking impedes the ability to solve complex intelligence tasks (Jensen 1992; Joly-Burra et al. 2018; Rammsayer and Troche 2010; Schmiedek et al. 2007; Schulz-Zhecheva et al. 2016). Theoretically, greater intraindividual RT variability represents transient fluctuations in behavioral performance, and has been linked with attentional lapses and fluctuating executive control (West et al. 2002). Neuroimaging research supports the idea that RT variability is an indicator of lower neurobiological integrity, including reduced white matter volume (Jackson et al. 2012) and increased white matter hyperintensity volume (Bunce et al. 2007). Lifespan cognitive research has further suggested that age-related dopamine depletion reduces the neural signal-to-noise ratio, such that more intermittent brain signaling leads to more behavioral variability and reductions in a wide range of cognitive abilities (MacDonald et al. 2012). Out of the three RT components from the expanded location-scale model, this component had the weakest associations with cognitive test scores, and the least durable prospective associations in lagged analyses. One possible explanation is that even after removing intraindividual RT variation in relation to differences in the items’ TIs, the residual RT variability component did not consist of purely spontaneous variability (Type 1 variability in Fiske and Rice’s terminology) in RTs.

Exploratory analyses examining age differences in the relationships between the RT components and cognitive test scores showed that slower mean RTs were more strongly associated with worse performance on all three cognitive tests for older compared to younger participants. Prior psychometric work has indicated that associations between performance across various cognitive and sensory tasks generally strengthen over the adult lifespan, suggesting that the structure of individuals’ cognitive abilities becomes less differentiated in older age (Hülür et al. 2015; Li et al. 2004). While speculative, our finding that mean RTs in surveys relate more strongly to inductive reasoning and task switching/inhibitory control at older compared to younger ages is perhaps consistent with this dedifferentiation hypothesis of cognitive aging. Previous research has also shown that survey satisficing and insufficient effort responding are more prevalent at younger compared to older ages (Schneider et al. 2018); thus, it is also possible that insufficient effort responding (which can manifest in fast RTs in survey responding, Bowling et al. forthcoming) among younger participants diluted the relationships with cognitive test scores in this age group.

### 4.3. Implications for Research

To date, survey research has predominantly utilized RTs for evaluating the quality of survey questions (Yan and Tourangeau 2008), detecting careless responding and survey fatigue (Galesic and Bosnjak 2009; Meade and Craig 2012; Schneider et al. 2018), and studying attitude strength (Fazio 1990). The results of the present study have implications for cognitive functioning research in that the scope of RT applications could be broadened to aid in the monitoring of cognitive abilities in large population surveys. With the increase in web-based data collection, RTs are now routinely assessed and have become a readily available byproduct in most online studies. Our study using more than 5 million RTs from survey items in a nationally representative sample demonstrates the potential of harnessing this type of data and corroborates the feasibility of utilizing survey item RTs for intelligence research. Our longitudinal (lagged) results further illustrate potential implications for epidemiology and health policy research in that they suggest avenues for the prospective prediction of cognitive abilities in survey studies when standard cognitive tests are not available.

Our findings also have implications for web-based survey design. Even though survey researchers are well aware of the response burden inherent in completing lengthy questionnaires, relatively little research has examined the linkages between survey response burden and participants’ level of cognitive ability in general population surveys. Our evidence that lower intellectual abilities are systematically related to longer RTs in survey items, strongly suggests that lower intellectual ability is associated with greater respondent burden as it will take people with lower cognitive abilities longer to complete a questionnaire. The cumulative effects of longer RTs may result in survey fatigue, especially in these individuals. Moreover, for survey development care should be taken when considering the nature and demands of survey items on the respondent. Surveys consisting of very heterogeneous sets of items place greater demand on respondents in that they need to adjust their attentional focus and adapt their responses as they navigate through different sets of questions. This might be particularly challenging for respondents with lower cognitive abilities and could potentially lead to greater attrition and missing data, particularly for repeated assessments of the same survey.

### 4.4. Limitations

Several study limitations should be noted. First, even though the cognitive tests examined in this study had been specifically developed for online administration (Liu et al. 2022; McArdle et al. 2015), they were self-administered and completed on the web at participants’ own convenience, which reduces the level of standardization often seen in tests administered by trained professionals in precisely controlled test environments.

Similarly, participants completed the web-based surveys at their convenience, and the RTs recorded as paradata may be impacted by many environmental influences that were uncontrolled, including participants’ current location, the time of day on which a survey was completed, differences in the device used when completing the questions, and momentary environmental distractions.

We also assumed that the contents of the particular surveys and the domain specific demands posed by an item did not affect the results. The extent to which a given question poses higher or lower domain specific demands may likely be individual-specific in that it will depend, among other things, upon the person’s familiarity with the specific content domains tapped by the questions. That is, a respondent may find those questions particularly challenging to answer that tap issues and topics that they have limited experience with, compared to topics they are deeply familiar with. For example, individuals who follow politics closely may already have well-formed opinions about political issues, and therefore, they may respond quicker than individuals who do not follow politics closely. The same holds true for other topics. In this study, we have used items covering a broad range of topics, which should average out such interactions between item content and individual characteristics, mitigating this concern. In future research with more clearly defined sets of items, one potential research strategy could be to assess participants’ familiarity with different item domains (e.g., in a separate set of self-report items) and to control RTs for participants’ familiarity with content domains at the item- and person-level.

Furthermore, there are additional aspects of intraindividual RT variability that could potentially be isolated from survey RT paradata and were not examined here, such as parameters of descriptive (e.g., ex-Gaussian, shifted Wald) RT distributions (Matzke and Wagenmakers 2009; Schmiedek et al. 2007). Even though these parameters have been linked to intelligence (Schmiedek et al. 2007), they are arguably most meaningfully derived from relatively homogeneous reaction time tasks involving quick decision making, and we speculate that they may not be well suited for capturing cognitive processes in responses to heterogeneous questionnaire items. As another example, Joly-Burra et al. (2018) applied dynamic structural equation modeling to reaction times in a classical Go/NoGo task to measure “coherence” in a person’s RT pattern (in addition to measures of mean RT and RT variability), operationalized as the autocorrelation of consecutive RTs (i.e., the extent to which momentary deviations from a person’s mean RT carry over to the next item). The expanded location-scale model used in the present study could be further expanded to incorporate individual-specific autoregressive effects in RTs (i.e., to measure “coherence” in RTs). However, this model would benefit from having response times for consecutive survey items available, which was not the case in our study because RTs of items administered together on a page (presented in grid or matrix format) were not time-stamped separately as paradata and could not be included in the analyses.

Finally, it should be kept in mind that even though we used a wide range of survey items, the predictions may not generalize to all survey items and content domains, and they were derived from a single internet panel. Our results require replication using RT paradata in other online survey studies.

## 5. Conclusions

The present study found that RTs to online items in survey research have the potential to provide information about people’s cognitive abilities, including inductive reasoning skills and task switching/inhibitory control. Even though the amount of variance shared between the RT components and cognitive test scores was nowhere near values that would suggest that RTs from surveys can be used as a replacement of formal cognitive testing, our findings demonstrate the utility of harnessing this type of readily available paradata for intelligence research and open the door to innovative ways to approach the measurement of cognitive abilities on a population level. Our longitudinal (lagged) results further illustrate that RTs in survey research may be useful for the prospective prediction of cognitive abilities. We found that the expanded location-scale model outperformed the original multilevel location-scale model in the prediction of cognitive abilities by separating item-to-item RT variability into two distinct components that capture a person’s systematic RT adjustments to changing time intensities of the items and residual intraindividual RT variability addressing random noise in responding. Whereas the pool of survey items selected for the present study was heterogeneous in nature, future research could benefit from examining sets of survey items that differ in well-defined domains and cognitive demand characteristics; this would create additional opportunities for studying how item contents and demands may be differentially related to specific cognitive processing domains. We encourage future population and cognitive research to continue to investigate the multiple uses of survey item RTs and hope that the potential theoretical and empirical benefits of applying an expanded version of the location-scale model for understanding respondents’ cognitive abilities will continue to be further explored.

## Figures and Tables

**Table 1:** Demographic characteristics of the analysis sample for each cognitive test.

	Number Series, n (%) (n = 8772)	Verbal Analogies, n (%) (n = 8947)	Stop-and-Go Switching, n (%) (n = 6509)
Age (years)			
18–34	1810 (20.63)	1855 (20.73)	1363 (20.94)
35–44	1808 (20.61)	1832 (20.48)	1374 (21.11)
45–54	1580 (18.01)	1581 (17.67)	1196 (18.37)
55–64	1715 (19.55)	1755 (19.62)	1239 (19.04)
65–74	1300 (14.82)	1347 (15.06)	1002 (15.39)
>74	559 (6.37)	577 (6.45)	335 (5.15)
Gender			
Men	3607 (41.12)	3699 (41.34)	2639 (40.56)
Women	5165 (58.88)	5248 (58.66)	3867 (59.44)
Race/Ethnicity			
Non-Hispanic white	5580 (63.61)	5774 (64.55)	4242 (65.17)
Hispanic	1558 (17.78)	1492 (16.68)	1106 (15.46)
Other/mixed	1632 (18.60)	1679 (18.77)	1256 (19.30)
Education			
High school graduate or less	2041 (23.27)	2050 (22.92)	1311 (20.16)
More than high school/less than college graduate	3285 (37.45)	3368 (37.65)	2358 (36.25)
College graduate	3445 (39.28)	3528 (39.44)	2835 (43.59)
Annual Household Income			
Less than $25,000	1806 (20.64)	1829 (20.49)	1172 (18.05)
$25,000–$49,999	1928 (22.04)	1972 (22.09)	1378 (21.23)
$50,000–$74,999	1630 (18.63)	1668 (18.68)	1233 (18.99)
$75,000–$99,999	1166 (13.33)	1206 (13.51)	906 (13.96)
$100,000 or more	2219 (25.36)	2253 (25.24)	1803 (27.77)

Note: Some frequencies sum to less than the total sample size due to missing values.

**Table 2:** Relationships (correlations and standardized regression coefficients) between cognitive test scores and survey item response time components derived from the location-scale model.

	Correlations		Multiple Regressions				
	*r*	*p*	β	*t*	*F*	R^2^	*p*
Outcome: Number Series							
Without demographic covariates					192.00 ^a^	.04	<.001
Mean RT	−.06	<.001	−.06	−6.04			<.001
RT variability	.19	<.001	.19	18.45			<.001
With demographic covariates					507.68 ^b^	.30	<.001
Mean RT			−.01	−0.51			.61
RT variability			.20	21.82			<.001
Outcome: Verbal Analogies							
Without demographic covariates					225.30 ^c^	.05	<.001
Mean RT	−.17	<.001	−.16	−16.07			<.001
RT variability	.14	<.001	.14	13.32			<.001
With demographic covariate					362.29 ^d^	.24	<.001
Mean RT			−.08	−7.25			<.001
RT variability			.12	12.04			<.001
Outcome: Stop-and-Go Switching							
Without demographic covariates					759.82 ^e^	.19	<.001
Mean RT	−.41	<.001	−.41	−36.62			<.001
RT variability	.15	<.001	.14	12.65			<.001
With demographic covariates					269.72 ^f^	.25	<.001
Mean RT			−.31	−3.05			<.001
RT variability			.10	8.87			<.001

Note: Regression coefficients for models with demographic covariates are statistically controlled for age, gender, race, ethnicity, education, and income. ^a^ *df* = 2, 8769; ^b^ *df* = 8, 8734; ^c^ *df* = 2, 8944; ^d^ *df* = 8, 8913; ^e^ *df* = 2, 6506; ^f^ *df* = 8, 6464.

**Table 3:** Age differences in relationships between cognitive test scores and survey item response time components derived from the location-scale model.

	Less than 40 Years of Age			40+ Years of Age			Age Difference in Relationships	
	β	*t*	*p*	β	*t*	*p*	*t*	*p*
Number Series F_5, 8766_ = 94.39, R^2^ = .05								
Mean RT	.03	1.68	.09	−.17	−10.61	<.001	−11.68	<.001
RT variability	.17	9.22	<.001	.23	16.30	<.001	2.71	.01
Verbal Analogies F_5, 8941_ = 127.13, R^2^ = .06								
Mean RT	.01	.37	.71	−.32	−20.03	<.001	−13.20	<.001
RT variability	.12	6.47	<.001	.16	11.44	<.001	1.67	.09
Stop-and-Go Switch F_5, 6503_ = 364.23, R^2^ = .22								
Mean RT	−.25	−14.47	<.001	−.48	−23.81	<.001	−8.72	<.001
RT variability	.08	4.97	<.001	.12	6.90	<.001	1.88	.06

**Table 4:** Relationships (correlations and standardized regression coefficients) between cognitive test scores and survey item response time components derived from the expanded location-scale model.

	Correlations		Multiple Regressions				
	*r*	*p*	β	*t*	*F*	R^2^	*p*
Outcome: Number Series							
Without demographic covariates					1095.44 ^a^	.26	<.001
Mean RT	−.09	<.001	−.28	−28.69			<.001
Systematic RT adjustments	.43	<.001	.50	52.76			<.001
Residual RT variability	−.12	<.001	−.15	−16.06			<.001
With demographic covariates					646.96 ^b^	.39	<.001
Mean RT			−.16	−14.99			<.001
Systematic RT adjustments			.39	41.73			<.001
Residual RT variability			−.05	−5.13			<.001
Outcome: Verbal Analogies							
Without demographic covariates					877.73 ^c^	.22	<.001
Mean RT	−.20	<.001	−.36	−35.88			<.001
Systematic RT adjustments	.33	<.001	.42	43.38			<.001
Residual RT variability	−.10	<.001	−.16	−16.58			<.001
With demographic covariates					456.76 ^d^	.31	<.001
Mean RT			−.22	−19.48			<.001
Systematic RT adjustments			.31	31.25			<.001
Residual RT variability			−.09	−9.10			<.001
Outcome: Stop-and-go Switching							
Without demographic covariates					596.59 ^e^	.22	<.001
Mean RT	−.41	<.001	−.48	−39.47			<.001
Systematic RT adjustments	.05	<.001	.22	18.60			<.001
Residual RT variability	.16	<.001	.04	3.43			<.001
With demographic covariates					257.38 ^f^	.26	<.001
Mean RT			−.32	−22.81			<.001
Systematic RT adjustments			.16	13.10			<.001
Residual RT variability			−.04	−2.96			<.001

Note: Regression coefficients for models with demographic covariates are statistically controlled for age, gender, race, ethnicity, education, and income. ^a^ *df* = 3, 8768; ^b^ *df* = 9, 8733; ^c^ *df* = 3, 8943; ^d^ *df* = 9, 8912; ^e^ *df* = 3, 6505; ^f^ *df* = 9, 6463.

**Table 5:** Age differences in relationships between cognitive test scores and survey item response time components derived from the expanded location-scale model.

	Less than 40 Years of Age			40+ Years of Age			Age Difference in Relationships	
	β	*t*	*p*	β	*t*	*p*	*t*	*p*
Number Series F_7, 8764_ = 485.48, R^2^ = .26								
Mean RT	−.20	−6.74	<.001	−.30	−26.36	<.001	−3.07	.002
Systematic RT adjustments	.57	24.67	<.001	.51	44.75	<.001		.05
Residual RT variability	−.12	−4.90	<.001	−.17	−14.39	<.001		.13
Verbal Analogies F_7, 8939_ = 385.24, R^2^ = .23								
Mean RT	−.19	−6.27	<.001	−.39	−33.88	<.001		<.001
Systematic RT adjustments	.44	18.30	<.001	.43	37.24	<.001		.59
Residual RT variability	−.16	−6.10	<.001	−17	−14.59	<.001	−.36	.72
Stop-and-Go Switch F_7, 6503_ = 287.58, R^2^ = .24								
Mean RT	−.25	−9.45	<.001	−.53	−33.80	<.001	−9.04	<.001
Systematic RT adjustments	.16	7.88	<.001	.22	14.01	<.001	1.93	.05
Residual RT variability	−.03	−1.48	.14	.02	1.08	.28	1.83	.07

**Table 6:** Results for response time components as predictors of number series scores by time lag between surveys and cognitive assessment.

		Correlations						Multiple Regression ^a^					
		Mean RT		Systematic RT Adjustment		Residual RT Variability		Mean RT		Systematic RT Adjustments		Residual RT Variability	
Years of Surveys before Number Series	*n*	*r*	*p*	*r*	*p*	*r*	*p*	β (*t*-Value)	*p*	β (*t*-Value)	*p*	β (*t*-Value)	*p*
0 to 0.5	8198	−.10	<.001	.35	<.001	−.15	<.001	−.30 (−20.66)	<.001	.57 (32.49)	<.001	−.20 (−11.94)	<.001
>0.5 to 1	7578	−.07	<.001	.17	<.001	−.12	<.001	−.21 (−13.10)	<.001	.35 (16.89)	<.001	−.15 (−12.92)	<.001
>1 to 1.5	5039	−.07	<.001	.18	<.001	−.12	<.001	−.21 (−11.44)	<.001	.35 (13.99)	<.001	−.17 (−9.72)	<.001
>1.5 to 2	6901	−.11	<.001	.28	<.001	−.12	<.001	−.33 (−20.13)	<.001	.56 (26.09)	<.001	−.16 (−9.22)	<.001
>2 to 2.5	7421	−.08	<.001	.34	<.001	−.06	<.001	−.25 (−16.90)	<.001	.48 (30.90)	<.001	−.07 (−4.73)	<.001
>2.5 to 3	4455	−.07	<.001	.28	<.001	−.04	.05	−.22 (−11.85)	<.001	.39 (19.86)	<.001	−.06 (−3.42)	<.001
>3 to 3.5	3529	−.09	<.001	.17	<.001	−.10	<.001	−.27 (−11.17)	<.001	.35 (13.70)	<.001	−.07 (−3.67)	<.001
>3.5 to 4	4290	−.11	<.001	.29	<.001	−.10	<.001	−.36 (−16.04)	<.001	.52 (19.72)	<.001	−.03 (−1.23)	.22
>4 to 4.5	4199	−.08	<.001	.31	<.001	−.04	.02	−.18 (−9.09)	<.001	.31 (17.50)	<.001	−.05 (−3.03)	.003
>4.5 to 5	1727	−.12	<.001	.34	<.001	−.04	.20	−.35 (−10.74)	<.001	.44 (14.17)	<.001	−.04 (−1.29)	.20
>5 to 5.5	1275	−.15	<.001	.28	<.001	−.15	<.001	−.50 (−10.31)	<.001	.51 (11.67)	<.001	−.02 (−0.58)	.61
>5.5 to 6	1096	−.13	<.001	.27	<.001	−.05	.27	−.40 (−9.83)	<.001	.38 (8.79)	<.001	−.02 (−0.50)	.61
>6 to 6.5	693	−.10	.01	.18	<.001	.11	.03	−.31 (−5.89)	<.001	.17 (4.57)	<.001	.08 (1.61)	.13

Note: ^a^ Overall model fit: F_51, 8771_ = 40.71 (*p* < .001); mean RT: F_1, 8771_ = 348.59 (*p* < .001); systematic RT adjustments: F_1, 8771_ = 957.76 (*p* < .001); residual RT variability: F_1, 8771_ = 40.39 (*p* < .001); main effect for time: F_12, 8771_ = 7.04 (*p* < .001); mean RT by lag period interaction: F_12, 8771_ = 18.78 (*p* < .001); systematic RT adjustments by lag period interaction: F_12, 8771_ = 27.76 (*p* < .001); residual RT variability by lag period interaction: F_12, 8771_ = 11.20 (*p* < .001).

**Table 7:** Results for response time components as predictors of verbal analogies scores by time lag between surveys and cognitive assessment.

		Correlations						Multiple regression ^a^					
		Mean RT		Systematic RT Adjustment		Residual RT Variability		Mean RT		Systematic RT Adjustments		Residual RT Variability	
Years of Surveys before Verbal Analogies	*n*	*r*	*p*	*r*	*p*	*r*	*p*	β (*t*-Value)	*p*	β (*t*-Value)	*p*	β (*t*-Value)	*p*
0 to 0.5	8646	−.23	<.001	.25	<.001	−.08	<.001	−.36 (−23.82)	<.001	.52 (27.63)	<.001	−.15 (−10.00)	<.001
>0.5 to 1	7125	−.17	<.001	.10	<.001	−.09	<.001	−.27 (−16.80)	<.001	.33 (14.89)	<.001	−.14 (−8.19)	<.001
>1 to 1.5	4013	−.18	<.001	.11	<.001	−.09	<.001	−.28 (−12.87)	<.001	.35 (11.67)	<.001	−.14 (−6.50)	<.001
>1.5 to 2	6614	−.22	<.001	.19	<.001	−.09	<.001	−.37 (−20.56)	<.001	.50 (20.24)	<.001	−.04 (−7.58)	<.001
>2 to 2.5	7131	−.19	<.001	.24	<.001	−.09	<.001	−.32 (−19.57)	<.001	.46 (23.92)	<.001	−.12 (−7.39)	<.001
>2.5 to 3	4596	−.22	<.001	.20	<.001	−.01	.46	−.28 (−15.83)	<.001	.34 (16.64)	<.001	−.05 (−2.79)	.005
>3 to 3.5	3331	−.17	<.001	.17	<.001	−.06	.002	−.31 (−13.81)	<.001	.30 (12.05)	<.001	−.03 (−1.67)	.10
>3.5 to 4	4180	−.19	<.001	.12	<.001	−.07	<.001	−.36 (−16.58)	<.001	.39 (14.59)	<.001	−.01 (−0.65)	.52
>4 to 4.5	3843	−.15	<.001	.17	<.001	.01	.62	−.27 (−12.48)	<.001	.24 (11.22)	<.001	−.02 (−0.96)	.34
>4.5 to 5	1827	−.17	<.001	.19	<.001	−.04	.14	−.27 (−8.75)	<.001	.29 (8.95)	<.001	−.04 (−1.57)	.12
>5 to 5.5	1305	−.30	<.001	.25	<.001	−.05	.15	−.50 (−11.02)	<.001	.39 (9.26)	<.001	.05 (−1.43)	.15
>5.5 to 6	1099	−.25	<.001	.15	<.001	−.11	.004	−.38 (−9.40)	<.001	.21 (5.27)	<.001	−.05 (−1.27)	.21
>6 to 6.5	752	−.21	<.001	.09	.02	.01	.77	−.33 (−6.59)	<.001	.08 (2.16)	.03	.03 (0.69)	.49

Note: ^a^ Overall model fit: F_51, 8946_ = 30.55 (*p* < .001); mean RT: F_1, 8946_ = 440.74 (*p* < .001); systematic RT adjustments: F_1, 8946_ = 599.22 (*p* < .001); residual RT variability: F_1, 8946_ = 26.29 (*p* < .001); main effect for time: F_12, 8946_ = 2.90 (*p* < .001); mean RT by lag period interaction: F_12, 8946_ = 9.90 (*p* < .001); systematic RT adjustments by lag period interaction: F_12, 8946_ = 25.00 (*p* < .001); residual RT variability by lag period interaction: F_12, 8946_ = 9.58 (*p* < .001).

**Table 8:** Results for response time components as predictors of stop-and-go switch task scores by time lag between surveys and cognitive assessment.

		Correlations						Multiple Regression ^a^					
		Mean RT		Systematic RT Adjustment		Residual RT Variability		Mean RT		Systematic RT Adjustments		Residual RT Variability	
Years of Surveys before Stop-and-Go Task	*n*	*r*	*p*	*r*	*p*	*r*	*p*	β (*t*-Value)	*p*	β (*t*-Value)	*p*	β (*t*-value)	*p*
0 to 0.5	6351	−.28	<.001	.14	<.001	.17	<.001	−.37 (−18.29)	<.001	.19 (7.48)	<.001	−.07 (−4.26)	<.001
>0.5 to 1	5984	−.29	<.001	.12	<.001	.17	<.001	−.38 (−18.01)	<.001	.18 (10.02)	<.001	−.07 (−3.75)	<.001
>1 to 1.5	5238	−.25	<.001	.10	<.001	.13	.002	−.32 (−14.59)	<.001	.14 (6.39)	<.001	−.02 (−1.06)	.29
>1.5 to 2	2969	−.24	<.001	.13	<.001	.14	.02	−.33 (−12.30)	<.001	.18 (7.70)	<.001	−.04 (−1.97)	.05
>2 to 2.5	4171	−.27	<.001	.14	<.001	.15	<.001	−.38 (−16.26)	<.001	.21 (9.60)	<.001	−.06 (−2.72)	.01
>2.5 to 3	3910	−.25	<.001	.08	<.001	.18	.68	−.31 (−14.57)	<.001	.13 (7.10)	<.001	.00 (0.13)	.90
>3 to 3.5	2915	−.27	<.001	.08	<.001	.10	<.001	−.32 (−11.83)	<.001	.12 (6.08)	<.001	−.00 (−0.16)	.87
>3.5 to 4	2910	−.30	<.001	.06	.002	.05	.02	−.36 (−12.07)	<.001	.11 (4.75)	<.001	.01 (0.54)	.59
>4 to 4.5	2902	−.34	<.001	.07	<.001	.07	<.001	−.39 (−11.81)	<.001	.12 (4.71)	<.001	.01 (0.60)	.55
>4.5 to 5	2274	−.27	<.001	.01	.65	.06	.002	−.28 (−8.87)	<.001	.03 (1.51)	.13	.05 (2.58)	.01
>5 to 5.5	1218	−.38	<.001	.02	.43	.05	.11	−.40 (−8.45)	<.001	.06 (1.62)	.11	.03 (0.98)	.33
>5.5 to 6	916	−.49	<.001	.04	.22	.05	.10	−.57 (−8.30)	<.001	.12 (2.34)	.02	.10 (2.19)	.03
>6 to 6.5	826	−.42	.01	−.01	.77	−.02	.57	−.43 (−7.38)	<.001	.02 (0.33)	.74	.04 (0.76)	.45

Note: ^a^ Overall model fit: F_51, 6508_ = 28.23 (*p* < .001); mean RT: F_1, 6508_ = 227.50 (*p* < .001); systematic RT adjustments: F_1, 6508_ = 84.03 (*p* < .001); residual RT variability: F_1, 6508_ = .00 (*p* = .99); main effect for time: F_12, 6508_ = 2.60 (*p* = .002); mean RT by lag period interaction: F_12, 6508_ = 6.73 (*p* < .001); systematic RT adjustments by lag period interaction: F_12, 6508_ = 5.53 (*p* < .001); residual RT variability by lag period interaction: F_12, 6508_ = 3.76 (*p* < .001).

## Data Availability

Data are available at https://uasdata.usc.edu/.

## Supplementary Materials

The following supporting information can be downloaded at: https://www.mdpi.com/article/10.3390/jintelligence11010003/s1, Table S1: A sample of 50 survey items in the Understanding America Study used for the measurement of survey item response times. Figure S1: Path diagram of the multilevel structural equation model used to estimate the parameters of the expanded version of a “location-scale” multilevel model. Figure S2: Mplus code of the multilevel structural equation model to decompose the item response time data into three latent between-person components using the expanded version of the multilevel location scale model. Figure S3: Standardized regression coefficients for the prediction of cognitive test scores from time-lagged survey item response time (RT) components derived from the naive location-scale model. Figure S4: Standardized regression coefficients for the prediction of cognitive test scores from time-lagged survey item response time (RT) components derived from the naive location-scale model. Regression coefficients control for age, gender, race, ethnicity, education, and income. Figure S5: Standardized regression coefficients for the prediction of cognitive test scores from time-lagged survey item response time (RT) components derived from the expanded location-scale model.

## References

[B1-jintelligence-11-00003] Alattar Laith, Messel Matt, Rogofsky David (2018). An introduction to the Understanding America Study Internet panel. Social Security Bulletin.

[B2-jintelligence-11-00003] Batty G. David, Deary Ian J., Gottfredson Linda S. (2007). Premorbid (early life) IQ and later mortality risk: Systematic review. Annals of Epidemiology.

[B3-jintelligence-11-00003] Bissig David, Kaye Jeffrey, Erten-Lyons Deniz (2020). Validation of SATURN, a free, electronic, self-administered cognitive screening test. Alzheimer’s & Dementia: Translational Research & Clinical Interventions.

[B4-jintelligence-11-00003] Bowling Nathan A., Huang Jason L., Brower Cheyna K., Bragg Caleb B. The quick and the careless: The construct validity of page time as a measure of insufficient effort responding to surveys. Organizational Research Methods.

[B5-jintelligence-11-00003] Bunce David, Anstey Kaarin J., Christensen Helen, Dear Keith, Wen Wei, Sachdev Perminder (2007). White matter hyperintensities and within-person variability in community-dwelling adults aged 60–64 years. Neuropsychologia.

[B6-jintelligence-11-00003] Castro-Lionard Karine, Thomas-Antérion Catherine, Crawford-Achour Emilie, Rouch Isabelle, Trombert-Paviot Béatrice, Barthélémy Jean-Claude, Laurent Bernard, Roche Frédéric, Gonthier Régis (2011). Can maintaining cognitive function at 65 years old predict successful ageing 6 years later? The PROOF study. Age Ageing.

[B7-jintelligence-11-00003] Clark Caron A. C., Pritchard Verena E., Woodward Lianne J. (2010). Preschool executive functioning abilities predict early mathematics achievement. Developmental Psychology.

[B8-jintelligence-11-00003] Cohen Jacob (1988). Statistical Power Analysis for the Behavioral Sciences.

[B9-jintelligence-11-00003] Coomans Frederik, Hofman Abe, Brinkhuis Matthieu, van der Maas Han L. J., Maris Gunter (2016). Distinguishing fast and slow processes in accuracy-response time data. PLoS ONE.

[B10-jintelligence-11-00003] Couper Mick P., Kapteyn Arie, Schonlau Matthias, Winter Joachim (2007). Noncoverage and nonresponse in an Internet survey. Social Science Research.

[B11-jintelligence-11-00003] Deary Ian J., Caryl Peter G. (1997). Neuroscience and human intelligence differences. Trends in Neurosciences.

[B12-jintelligence-11-00003] Dodonova Yulia A., Dodonov Yury S. (2013). Faster on easy items, more accurate on difficult ones: Cognitive ability and performance on a task of varying difficulty. Intelligence.

[B13-jintelligence-11-00003] Duff Kevin, Mold James W., Gidron Yori (2009). Cognitive functioning predicts survival in the elderly. Journal of Clinical and Experimental Neuropsychology.

[B14-jintelligence-11-00003] Enkvist Åsa, Ekström Henrik, Elmståhl Sölve (2013). Associations between cognitive abilities and life satisfaction in the oldest-old. Results from the longitudinal population study Good Aging in Skåne. Clinical Interventions in Aging.

[B15-jintelligence-11-00003] Evans Jonathan St B. T., Stanovich Keith E. (2013). Dual-process theories of higher cognition: Advancing the debate. Perspectives on Psychological Science.

[B16-jintelligence-11-00003] Fazio Russell H. (1990). Multiple processes by which attitudes guide behavior: The MODE model as an integrative framework. Advances in Experimental Social Psychology.

[B17-jintelligence-11-00003] Feenstra Heleen E. M., Vermeulen Ivar E., Murre Jaap M. J., Schagen Sanne B. (2018). Online self-administered cognitive testing using the Amsterdam cognition scan: Establishing psychometric properties and normative data. Journal of Medical Internet Research.

[B18-jintelligence-11-00003] Finkel Deborah, Reynolds Chandra A., McArdle John J., Gatz Margaret, Pedersen Nancy L. (2003). Latent growth curve analyses of accelerating decline in cognitive abilities in late adulthood. Developmental Psychology.

[B19-jintelligence-11-00003] Fiske Donald W., Rice Laura (1955). Intra-individual response variability. Psychological Bulletin.

[B20-jintelligence-11-00003] Furnham Adrian, Cheng Helen (2016). Childhood cognitive ability predicts adult financial well-being. Journal of Intelligence.

[B21-jintelligence-11-00003] Galesic Mirta, Bosnjak Michael (2009). Effects of questionnaire length on participation and indicators of response quality in a web survey. Public Opinion Quarterly.

[B22-jintelligence-11-00003] Gallassi Roberto, Morreale Angela, Di Sarro Rita, Lorusso Sebastiano (2002). Value of clinical data and neuropsychological measures in probable Alzheimer’s disease. Archives of Gerontology and Geriatrics.

[B23-jintelligence-11-00003] Gehring William J., Goss Brian, Coles Michael G. H., Meyer David E., Donchin Emanuel (1993). A neural system for error detection and compensation. Psychological Science.

[B24-jintelligence-11-00003] Goldhammer Frank, Entink Rinke H. Klein (2011). Speed of reasoning and its relation to reasoning ability. Intelligence.

[B25-jintelligence-11-00003] Goldhammer Frank, Naumann Johannes, Stelter Annette, Tóth Krisztina, Rölke Heiko, Klieme Eckhard (2014). The time on task effect in reading and problem solving is moderated by task difficulty and skill: Insights from a computer-based large-scale assessment. Journal of Educational Psychology.

[B26-jintelligence-11-00003] Hamaker Ellen L., Wichers Marieke (2017). No time like the present: Discovering the hidden dynamics in intensive longitudinal data. Current Directions in Psychological Science.

[B27-jintelligence-11-00003] Hanes Doug P., Schall Jeffrey D. (1996). Neural control of voluntary movement initiation. Science.

[B28-jintelligence-11-00003] Haynes Becky I., Bauermeister Sarah, Bunce David (2017). A systematic review of longitudinal associations between reaction time intraindividual variability and age-related cognitive decline or impairment, dementia, and mortality. Journal of the International Neuropsychological Society.

[B29-jintelligence-11-00003] Hedeker Donald, Mermelstein Robin J., Demirtas Hakan (2012). Modeling between-subject and within-subject variances in ecological momentary assessment data using mixed-effects location scale models. Statistics in Medicine.

[B30-jintelligence-11-00003] Hedge Craig, Vivian-Griffiths Solveiga, Powell Georgina, Bompas Aline, Sumner Petroc (2019). Slow and steady? Strategic adjustments in response caution are moderately reliable and correlate across tasks. Consciousness and Cognition.

[B31-jintelligence-11-00003] Holroyd Clay B., Coles Michael G. H. (2002). The neural basis of human error processing: Reinforcement learning, dopamine, and the error-related negativity. Psychological Review.

[B32-jintelligence-11-00003] Hughes Matthew L., Agrigoroaei Stefan, Jeon Minjeong, Bruzzese Molly, Lachman Margie E. (2018). Change in cognitive performance from midlife into old age: Findings from the Midlife in the United States (MIDUS) study. Journal of the International Neuropsychological Society.

[B33-jintelligence-11-00003] Hülür Gizem, Ram Nilam, Willis Sherry L., Schaie K. Warner, Gerstorf Denis (2015). Cognitive dedifferentiation with increasing age and proximity of death: Within-person evidence from the Seattle Longitudinal Study. Psychology and Aging.

[B34-jintelligence-11-00003] Hunt Earl (1980). Intelligence as an information-processing concept. British Journal of Psychology.

[B35-jintelligence-11-00003] Jackson Jonathan D., Balota David A., Duchek Janet M., Head Denise (2012). White matter integrity and reaction time intraindividual variability in healthy aging and early-stage Alzheimer disease. Neuropsychologia.

[B36-jintelligence-11-00003] Jensen Arthur R. (1992). The importance of intraindividual variation in reaction time. Personality and Individual Differences.

[B37-jintelligence-11-00003] Joly-Burra Emilie, Van der Linden Martial, Ghisletta Paolo (2018). Intraindividual variability in inhibition and prospective memory in healthy older adults: Insights from response regularity and rapidity. Journal of Intelligence.

[B38-jintelligence-11-00003] Kaye Jeffrey A., Maxwell Shoshana A., Mattek Nora, Hayes Tamara L., Dodge Hiroko, Pavel Misha, Jimison Holly B., Wild Katherine, Boise Linda, Zitzelberger Tracy A. (2011). Intelligent systems for assessing aging changes: Home-based, unobtrusive, and continuous assessment of aging. Journals of Gerontology Series B: Psychological Sciences and Social Sciences.

[B39-jintelligence-11-00003] Klein Entink Rinke H., Fox Jean-Paul, van der Linden Willem J. (2009). A multivariate multilevel approach to the modeling of accuracy and speed of test takers. Psychometrika.

[B40-jintelligence-11-00003] Kyllonen Patrick C., Zu Jiyun (2016). Use of response time for measuring cognitive ability. Journal of Intelligence.

[B41-jintelligence-11-00003] Lachman Margie E., Agrigoroaei Stefan, Tun Patricia A., Weaver Suzanne L. (2014). Monitoring cognitive functioning: Psychometric properties of the Brief Test of Adult Cognition by Telephone. Assessment.

[B42-jintelligence-11-00003] Langa Kenneth M., Llewellyn David J., Lang Iain A., Weir David R., Wallace Robert B., Kabeto Mohammed U., Huppert Felicia A. (2009). Cognitive health among older adults in the United States and in England. BMC Geriatrics.

[B43-jintelligence-11-00003] Lenzner Timo, Kaczmirek Lars, Lenzner Alwine (2010). Cognitive burden of survey questions and response times: A psycholinguistic experiment. Applied Cognitive Psychology.

[B44-jintelligence-11-00003] Li Shu-Chen, Lindenberger Ulman, Hommel Bernhard, Aschersleben Gisa, Prinz Wolfgang, Baltes Paul B. (2004). Transformations in the couplings among intellectual abilities and constituent cognitive processes across the life span. Psychological Science.

[B45-jintelligence-11-00003] Liu Ying, Schneider Stefan, Meijer Erik, Darling Jill, Orriens Bart, Gutsche Tania, Kapteyn Arie, Gatz Margaret (2022). Self-administered web-based tests of executive functioning and perceptual speed: Measurement development study with a large probability based survey panel. Journal of Medical Internet Research.

[B46-jintelligence-11-00003] Luciano Michelle, Marioni Riccardo E., Gow Alan J., Starr John M., Deary Ian J. (2009). Reverse causation in the association between C-reactive protein and fibrinogen levels and cognitive abilities in an aging sample. Psychosomatic Medicine.

[B47-jintelligence-11-00003] MacDonald Stuart W. S., Karlsson Sari, Rieckmann Anna, Nyberg Lars, Bäckman Lars (2012). Aging-related increases in behavioral variability: Relations to losses of dopamine D1 receptors. Journal of Neuroscience.

[B48-jintelligence-11-00003] Mather Nancy, Jaffe Lynne E. (2016). Woodcock-Johnson IV: Reports, Recommendations, and Strategies.

[B49-jintelligence-11-00003] Matzke Dora, Wagenmakers Eric-Jan (2009). Psychological interpretation of the ex-Gaussian and shifted Wald parameters: A diffusion model analysis. Psychonomic Bulletin & Review.

[B50-jintelligence-11-00003] McArdle John, Rodgers Willard, Willis Robert (2015). Cognition and Aging in the USA (CogUSA) 2007–2009.

[B51-jintelligence-11-00003] McKhann Guy M., Knopman David S., Chertkow Howard, Hyman Bradley T., Jack Clifford R., Kawas Claudia H., Klunk William E., Koroshetz Walter J., Manly Jennifer J., Mayeux Richard (2011). The diagnosis of dementia due to Alzheimer’s disease: Recommendations from the National Institute on Aging-Alzheimer’s Association workgroups on diagnostic guidelines for Alzheimer’s disease. Alzheimer’s & Dementia.

[B52-jintelligence-11-00003] McNeish Daniel, Hamaker Ellen L. (2020). A primer on two-level dynamic structural equation models for intensive longitudinal data in Mplus. Psychological Methods.

[B53-jintelligence-11-00003] Meade Adam W., Craig S. Bartholomew (2012). Identifying careless responses in survey data. Psychological Methods.

[B54-jintelligence-11-00003] Murnane Richard J., Willett John B., Duhaldeborde Yves, Tyler John H. (2000). How important are the cognitive skills of teenagers in predicting subsequent earnings?. Journal of Policy Analysis and Management.

[B55-jintelligence-11-00003] Muthén Linda K., Muthén Bengt O. (2017). Mplus: Statistical Analysis with Latent Variables: User’s Guide.

[B56-jintelligence-11-00003] Naumann Johannes (2019). The skilled, the knowledgeable, and the motivated: Investigating the strategic allocation of time on task in a computer-based assessment. Frontiers in Psychology.

[B57-jintelligence-11-00003] Naumann Johannes, Goldhammer Frank (2017). Time-on-task effects in digital reading are non-linear and moderated by persons’ skills and tasks’ demands. Learning and Individual Differences.

[B58-jintelligence-11-00003] Nye Christopher D., Ma Jingjing, Wee Serena (2022). Cognitive Ability and Job Performance: Meta-analytic Evidence for the Validity of Narrow Cognitive Abilities. Journal of Business and Psychology.

[B59-jintelligence-11-00003] Park Denise, Schwarz Nobert (2012). Cognitive Aging: A Primer.

[B60-jintelligence-11-00003] Rammsayer Thomas H., Troche Stefan J. (2010). Effects of age and the relationship between response time measures and psychometric intelligence in younger adults. Personality and Individual Differences.

[B61-jintelligence-11-00003] Ratcliff Roger (1993). Methods for dealing with reaction time outliers. Psychological Bulletin.

[B62-jintelligence-11-00003] Richter Tobias, Isberner Maj-Britt, Naumann Johannes, Kutzner Yvonne (2012). Prozessbezogene Diagnostik von Lesefähigkeiten bei Grundschulkindern. Zeitschrift für Pädagogische Psychologie.

[B63-jintelligence-11-00003] Robertson Kimberley Ferriman, Smeets Stijn, Lubinski David, Benbow Camilla P. (2010). Beyond the threshold hypothesis: Even among the gifted and top math/science graduate students, cognitive abilities, vocational interests, and lifestyle preferences matter for career choice, performance, and persistence. Current Directions in Psychological Science.

[B64-jintelligence-11-00003] Rutter Lauren A., Vahia Ipsit V., Forester Brent P., Ressler Kerry J., Germine Laura (2020). Heterogeneous indicators of cognitive performance and performance variability across the lifespan. Frontiers in Aging Neuroscience.

[B65-jintelligence-11-00003] Samejima Fumiko (1969). Estimation of Latent Ability Using a Response Pattern of Graded Scores. Psychometrika.

[B66-jintelligence-11-00003] Schagen Sanne B., Klein Martin, Reijneveld Jaap C., Brain Etienne, Deprez Sabine, Joly Florence, Scherwath Angela, Schrauwen Wim, Wefel Jeffrey S. (2014). Monitoring and optimising cognitive function in cancer patients: Present knowledge and future directions. European Journal of Cancer Supplements.

[B67-jintelligence-11-00003] Schmiedek Florian, Oberauer Klaus, Wilhelm Oliver, Süß Heinz-Martin, Wittmann Werner W. (2007). Individual differences in components of reaction time distributions and their relations to working memory and intelligence. Journal of Experimental Psychology: General.

[B68-jintelligence-11-00003] Schneider Stefan, Jin Haomiao, Orriens Bart, Junghaenel Doerte U., Kapteyn Arie, Meijer Erik, Stone Arthur A. Using Attributes of Survey Items to Predict Response Times May Benefit Survey Research. Field Methods.

[B69-jintelligence-11-00003] Schneider Stefan, May Marcella, Stone Arthur A. (2018). Careless responding in internet-based quality of life assessments. Quality of Life Research.

[B70-jintelligence-11-00003] Schulz-Zhecheva Yoanna, Voelkle Manuel C., Beauducel André, Biscaldi Monica, Klein Christoph (2016). Predicting fluid intelligence by components of reaction time distributions from simple choice reaction time tasks. Journal of Intelligence.

[B71-jintelligence-11-00003] Seelye Adriana, Hagler Stuart, Mattek Nora, Howieson Diane B., Wild Katherine, Dodge Hiroko H., Kaye Jeffrey A. (2015). Computer mouse movement patterns: A potential marker of mild cognitive impairment. Alzheimer’s & Dementia: Diagnosis, Assessment & Disease Monitoring.

[B72-jintelligence-11-00003] Selig James P., Preacher Kristopher J., Little Todd D. (2012). Modeling time-dependent association in longitudinal data: A lag as moderator approach. Multivariate Behavioral Research.

[B73-jintelligence-11-00003] Slifkin Andrew B., Newell Karl M. (1998). Is variability in human performance a reflection of system noise?. Current Directions in Psychological Science.

[B74-jintelligence-11-00003] St John Philip D., Montgomery Patrick R. (2010). Cognitive impairment and life satisfaction in older adults. International Journal of Geriatric Psychiatry.

[B75-jintelligence-11-00003] Stilley Carol S., Bender Catherine M., Dunbar-Jacob Jacqueline, Sereika Susan, Ryan Christopher M. (2010). The impact of cognitive function on medication management: Three studies. Health Psychology.

[B76-jintelligence-11-00003] Tam Angela, Luedke Angela C., Walsh Jeremy J., Fernandez-Ruiz Juan, Garcia Angeles (2015). Effects of reaction time variability and age on brain activity during Stroop task performance. Brain imaging and behavior.

[B77-jintelligence-11-00003] Thissen David, Weiss David J. (1983). Timed testing: An approach using item response theory. New Horizons in Testing.

[B78-jintelligence-11-00003] Tourangeau R., Rips L. J., Rasinski K. (2000). The Psychology of Survey Response.

[B79-jintelligence-11-00003] Tsoy Eduard, Zygouris Stelios, Possin Katherine L. (2021). Current state of self-administered brief computerized cognitive assessments for detection of cognitive disorders in older adults: A systematic review. The Journal of Prevention of Alzheimer’s Disease.

[B80-jintelligence-11-00003] Tun Patricia A., Lachman Margie E. (2008). Age differences in reaction time and attention in a national telephone sample of adults: Education, sex, and task complexity matter. Developmental Psychology.

[B81-jintelligence-11-00003] van der Linden Wim J. (2006). A lognormal model for response times on test items. Journal of Educational and Behavioral Statistics.

[B82-jintelligence-11-00003] Wai Jonathan, Brown Matt I., Chabris Christopher F. (2018). Using Standardized Test Scores to Include General Cognitive Ability in Education Research and Policy. Journal of Intelligence.

[B83-jintelligence-11-00003] West Robert, Murphy Kelly J., Armilio Maria L., Craik Fergus I. M., Stuss Donald T. (2002). Lapses of intention and performance variability reveal age-related increases in fluctuations of executive control. Brain and Cognition.

[B84-jintelligence-11-00003] Woodford Henry J, George James (2007). Cognitive assessment in the elderly: A review of clinical methods. QJM: An International Journal of Medicine.

[B85-jintelligence-11-00003] Yan Ting, Tourangeau Roger (2008). Fast times and easy questions: The effects of age, experience and question complexity on web survey response times. Applied Cognitive Psychology: The Official Journal of the Society for Applied Research in Memory and Cognition.

[B86-jintelligence-11-00003] Yeager David S., Krosnick Jon A., Chang LinChiat, Javitz Harold S., Levendusky Matthew S., Simpser Alberto, Wang Rui (2011). Comparing the accuracy of RDD telephone surveys and internet surveys conducted with probability and non-probability samples. Public Opinion Quarterly.

