# Supplementary material for: Inferring Cognitive Abilities from Response Times to Web-Administered Survey Items in a Population-Representative Sample
Source: J Intell. 2022 Dec 23;11(1):3. doi: 10.3390/jintelligence11010003 (PMC9864969; doi:10.3390/jintelligence11010003)

Supplementary

Inferring cognitive abilities from response times to web-administered survey items in a population-representative sample

Doerte U. Junghaenel, Stefan Schneider, Bart Orriens, Haomiao Jin, Pey-Jiuan Lee, Arie Kapteyn, Erik Meijer, Elizabeth M. Zelinski, Raymond Hernandez, Arthur A. Stone

Table S1. A sample of 50 survey items in the Understanding America Study used for the measurement of survey item response times.

• What is the percent chance that you will get the flu during the next year?
• Could you tell us how interesting or uninteresting you found the questions in this interview?
• Normally which asset described below displays the highest fluctuations over time: savings accounts bonds or stocks?
• On a scale of 1 to 10 how would you rate your neighborhood?
• Now thinking of all the buildings that are within a half block (or about 300 feet away) of your home do any of these buildings have metal bars on their windows?
• And what time did you go to sleep yesterday?
• Yesterday did you feel happy? Would you say:
• Are you currently working for pay?
• The following question asks you to rank your income in comparison with other people in your zip code. You can give a number between zero and one hundred. For instance if you believe you have the highest income in the zip code you enter 100. If you believe 60% of the people have higher incomes than you (and therefore 40% have less) you enter 40 etc.
• Overall how satisfied are you with the number of friends you have?
• Imagine that the interest rate on your savings account was 1% per year and inflation was 2% per year. After 1 year would you be able to buy more than exactly the same as or less than today with the money in this account?
• Do you think the following statement is true or false? "The cash value of a life insurance policy is the amount available if you surrender your life insurance policy while you're still alive."
• Have you ever contacted our Help Desk?
• In the last 3 years did you use any credit cards?
• Have you named someone to decide on your health care needs if you are unable to do it so yourself?
• Are you retired?
• Do you have any children?
• If a task is difficult it means that its important for me.
• Should we raise the smoking age to 21?
• When was your most recent crying episode?
• How good are you at working with percentages?
• Which of the following if any best describes your eating behavior?
• Over the past two weeks how often have you been bothered by the problem of feeling down depressed or hopeless?
• Have you ever personally experienced discrimination such as problems getting jobs housing education or services because of your race ethnicity religion sexual identity gender or country of origin?
• On what kind of device do you usually take our surveys?
• What do you think is the percent chance you will live to age 85?

• My Social Security is an online account that adults with a Social Security number can set up through the Social Security Administration. Have you previously heard about My Social Security?
• Some people believe that life forces or elements (for example: doshas yin and yang) have important effects on our health. Do you agree or disagree that balance between these life forces or elements is important to achieving good health?
• When you listen to music how often do you do you listen to music with others instead of listening to music alone?
• Overall how satisfied are you with your life?
• The system is stacked against people like me
• How important is being an American to who you are?
• Please think about the bills that you get regularly or every month (such as utility bills and your mortgage or rent). How many of your regular bills do you pay with automatic bill payment; that is having payments taken directly from your bank account by these companies every month without you having to schedule the payment? (please also count bills that are automatically charged to a credit card)
• Some people plan for someone else to make financial decisions for them if they become unable to make these decisions for themselves. They may make informal plans or they may sign a "power of attorney" which is a legal document that gives someone you choose the power to act in your place. Have you named someone who is authorized to make financial decisions for you?
• Do you have a mortgage?
• Are you married or do you have a partner who you live with?
• By "Incel" we mean someone who is unable to find a sexual partner despite desiring one. Do you identify as an Incel?
• From now until the election in November we would like to ask you a small number of questions every week about the 2016 Presidential election and your opinion about the candidates. We would ask you at most five simple questions each week and you would be paid $2 each week for your answers. Would you be interested in participating in this?
• What is the percent chance that you will vote in the November 2018 election for the U.S. House of Representatives?
• It bothers me when I hear immigrants speaking languages other than English in stores and restaurants in my community.
• Thinking of how you personally would define the terms "masculine" and "feminine" please move the slider on the bar below to represent how masculine or feminine you consider yourself to be. Click or tap on the bar then move the slider to where it should be
• To what extent do you agree or disagree with the following statement: All children no matter where they are born should be provided the opportunity to go to school develop their talents live safely and receive medical care when they need it.
• Suppose a person has two white grandparents and two Hispanic grandparents. Is that person best described as...
• In the past 30 days did you spend any time assisting a family member or close friend (e.g. parent grandparent wife husband adult child other family member neighbor or close friend) with their basic personal activities? By that we mean daily activities such as dressing eating bathing paying bills managing medication food preparation grocery shopping doctor visits emotional support driving and other types of personal assistance.
• Would you say that in general your physical health is:
• Do you currently have life insurance?
• Are you currently setting aside money for an emergency?
• On a scale of 0 to 100 what is the percent chance that a coronavirus vaccine will cause serious side effects or long-term health problems for someone who has been vaccinated? If you are unsure please give your best guess.
• Have you heard of the Antifa movement?
• Have you heard of the QAnon movement?

Figure S1. Path diagram of the multilevel structural equation model used to estimate the parameters of the expanded version of a “location-scale” multilevel model. The model is used to decompose the survey item response time (RT) data into three latent between-person components. At Level 1 (within-person), the observed (log-transformed) RTs are regressed on the observed time intensity for item *i*. Note that the values of TI were centered at 10 seconds, which means that the intercept captures the person’s predicted RT for an item with a time intensity of 10 seconds. the slope *s* captures the predicted increase and decrease in the person’s RTs for items with higher and lower time intensities (representing “systematic RT adjustments”). The residual variance σ^2 captures the deviations of the observed RTs from the predicted RTs (representing “residual RT variability”). Small filled circles at Level 1 indicate random effects that vary at Level 2 (between person). The intercept of RT and the regression slope s are random effects and vary between individuals. The residual variance σ^2 is also random and the log of the residual variance varies between individuals. The variances of the three parameters are latent variables (shown as circles) on the between-person level. Double headed arrows indicate that the latent variables are allowed to correlate with each other.

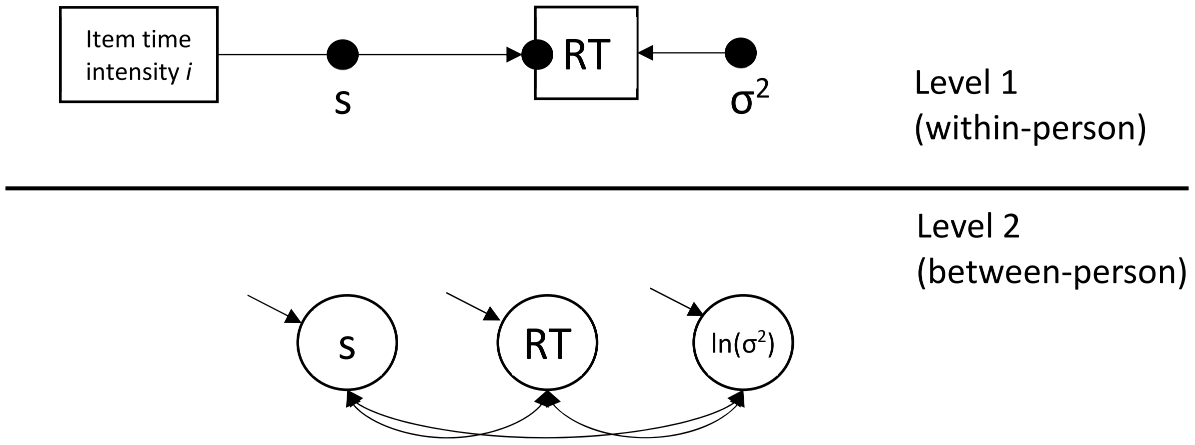

Figure S2. M*plus* code of the multilevel structural equation model to decompose the item response time data into three latent between-person components using the expanded version of the multilevel location scale model.

```
TITLE:
Expanded version of the multilevel location scale model for response times;
DATA:
FILE IS response_times.dat;          !response times data in “long” format
DEFINE:
TI = TI - log(10);                   !center the time intensity variable at the log of 10 seconds
VARIABLE:
NAMES ARE
id                                   !id variable
rt                                   !log-transformed response time variable
ti;                                  !indicator of the log-transformed item time-intensities
USEVARIABLES ARE
RT TI;
missing = .;                         !missing value flag
cluster = id;                        !declare id as clustering variable
within = ti;                         !declare TI as within-person variable
ANALYSIS:
TYPE IS TWOLEVEL RANDOM;             !two-level random-effects model
ESTIMATOR = BAYES;
BITER = (5000);                      !minimum number of Bayesian iterations set at 5000
MODEL:
%WITHIN%
s | rt ON ti;                        !regress response times on time intensities, with random slope s
resvar | rt;                         !estimate a random effect resvar for the residual variance
%BETWEEN%
[rt];                                !mean of random response time intercepts
[s];                                 !mean of random slopes s (“systematic RT adjustments”)
[resvar];                            !mean of log residual variance (“residual RT variability”)
rt s resvar  WITH rt s resvar;       !all three random effects are allowed to correlate with each other
OUTPUT:
TECH1 TECH8;
SAVEDATA:
FILE = estimates.txt;                !save the estimated latent factor scores for secondary analyses
SAVE = fscores (100 10);             !factor scores saved using 100 plausible values (factor 10 for thinning)
MISSFLAG = .;                        !missing value flag in saved dataset
```

Figure S3. Standardized regression coefficients for the prediction of cognitive test scores from time-lagged survey item response time (RT) components derived from the naive location-scale model. Error bars represent 95% confidence intervals.

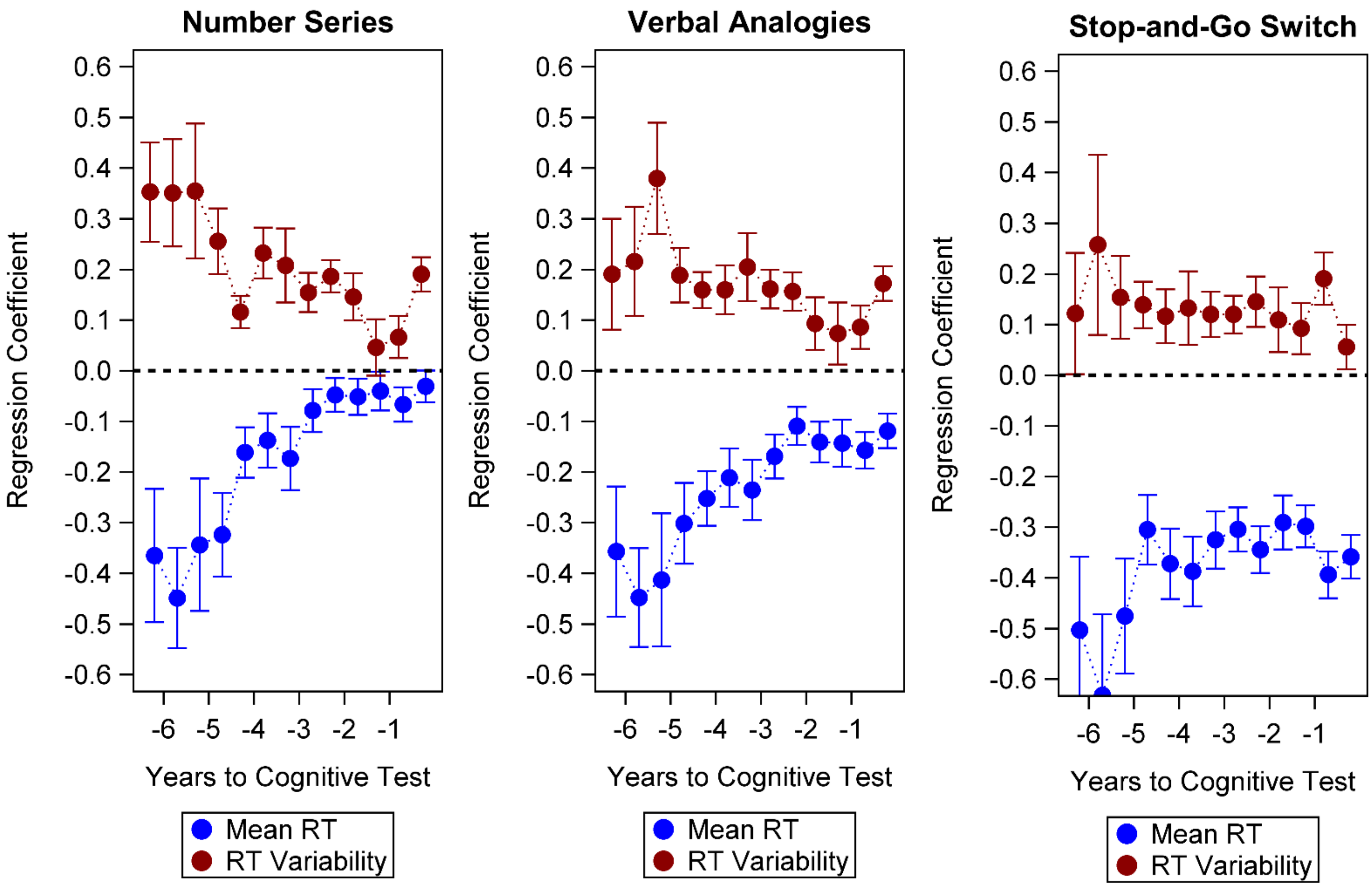

Figure S4. Standardized regression coefficients for the prediction of cognitive test scores from time-lagged survey item response time (RT) components derived from the naive location-scale model. Regression coefficients control for age, gender, race, ethnicity, education, and income. Error bars represent 95% confidence intervals.

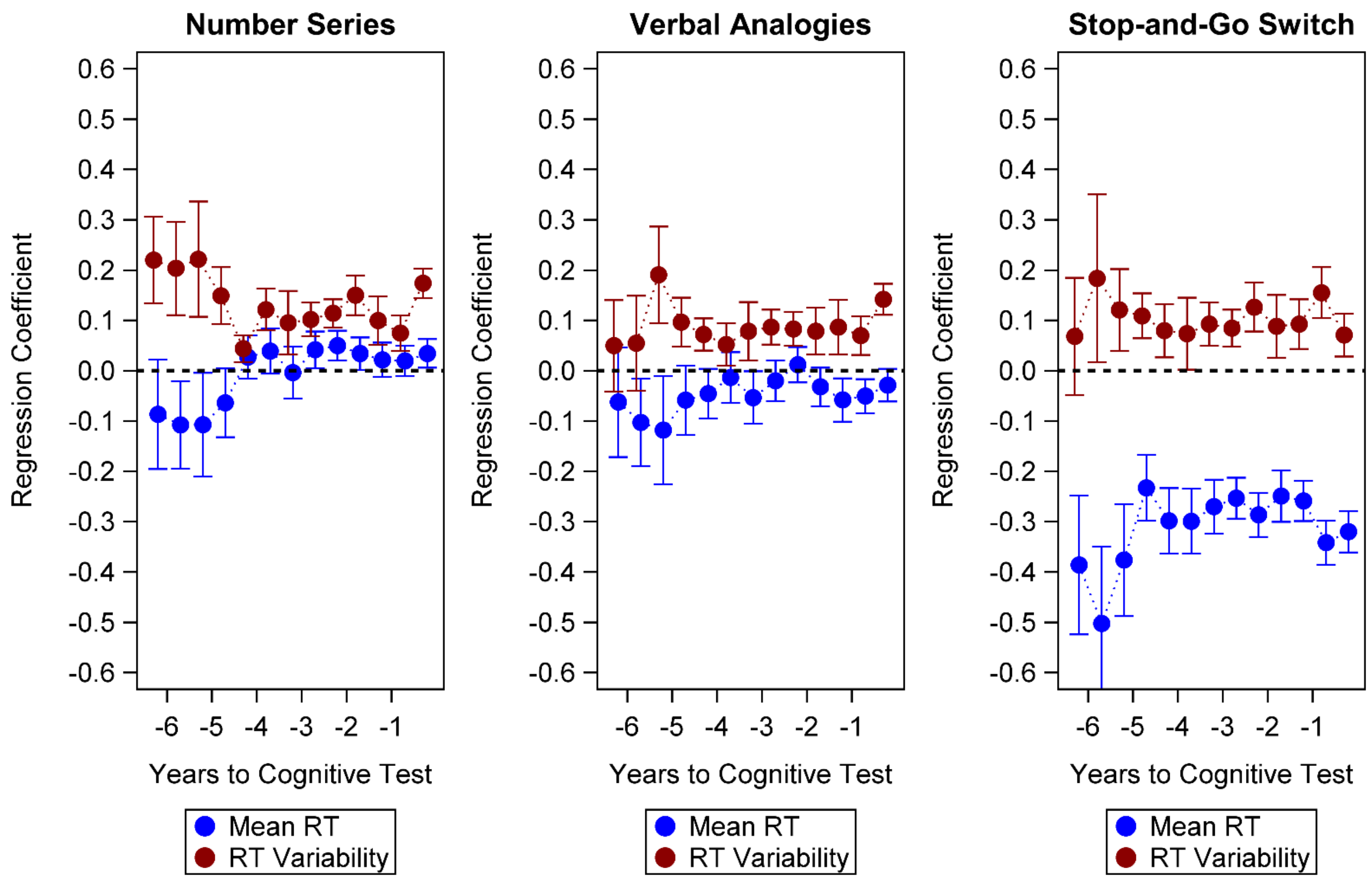

Figure S5. Standardized regression coefficients for the prediction of cognitive test scores from time-lagged survey item response time (RT) components derived from the expanded location-scale model. Regression coefficients control for age, gender, race, ethnicity, education, and income. Error bars represent 95% confidence intervals. Syst. = systematic; Res. = residual.

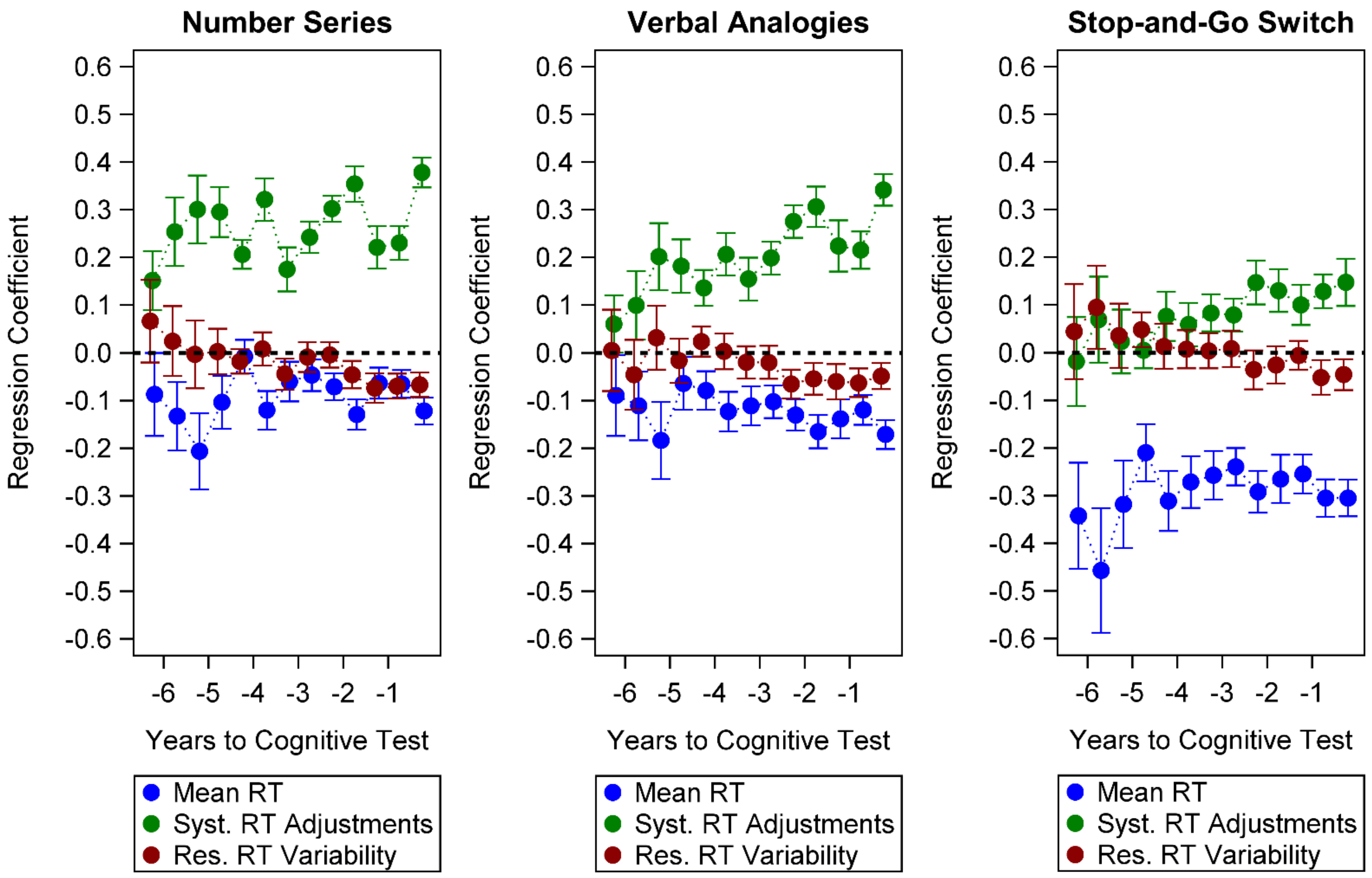

Supplement: Supplementary file 1 [file jintelligence-11-00003-s001.zip › jintelligence-2078688-supplementary.pdf]
